# Tight Complex Formation of the Fumarate Sensing DcuS-DcuR Two-Component System at the Membrane and Target Promoter Search by Free DcuR Diffusion

**DOI:** 10.1128/msphere.00235-22

**Published:** 2022-07-07

**Authors:** Stefaniya Gencheva, Simon Dersch, Kristin Surmann, Mathias Wernet, Luis Antelo, Elke Hammer, Peter L. Graumann, Nadja Hellmann, Gottfried Unden

**Affiliations:** a Institute for Molecular Physiology; Microbiology and Biotechnology, Johannes Gutenberg-University, Mainz, Germany; b Department of Chemistry, and Centre for Synthetic Microbiology, SYNMIKRO, Philipps-University Marburg, Marburg; c Department of Functional Genomics, Center for Functional Genomics of Microbes, University Medicine Greifswald, Greifswald, Germany; d IBWF gGmbH, Institute for Biotechnology and Drug Research, Mainz, Germany; e Department of Chemistry, Biochemistry, Johannes Gutenberg-University, Mainz, Germany; University of Iowa

**Keywords:** sensor kinase, two-component system, sensor complex, complex formation, bacteria, DcuS-DcuR

## Abstract

Signaling of two-component systems by phosphoryl transfer requires interaction of the sensor kinase with the response regulator. Interaction of the C4-dicarboxylate-responsive and membrane-integral sensor kinase DcuS with the response regulator DcuR was studied. *In vitro*, the cytoplasmic part of DcuS (PAS_C_-Kin) was employed. Stable complexes were formed, when either DcuS or DcuR were phosphorylated (*K_d_* 22 ± 11 and 28 ± 7 nM, respectively). The unphosphorylated proteins produced a more labile complex (*K_d_* 1380 ± 395 nM). Bacterial two-hybrid studies confirm interaction of DcuR with DcuS (and PAS_C_-Kin) *in vivo*. The absolute contents of DcuR (197-979 pmol mg^−1^ protein) in the bacteria exceeded those of DcuS by more than 1 order of magnitude. According to the *K_d_* values, DcuS exists in complex, with phosphorylated but also unphosphorylated DcuR. In live cell imaging, the predominantly freely diffusing DcuR becomes markedly less mobile after phosphorylation and activation of DcuS by fumarate. Portions of the low mobility fraction accumulated at the cell poles, the preferred location of DcuS, and other portions within the cell, representing phosphorylated DcuR bound to promoters. In the model, acitvation of DcuS increases the affinity toward DcuR, leading to DcuS-P × DcuR formation and phosphorylation of DcuR. The complex is stable enough for phosphate-transfer, but labile enough to allow exchange between DcuR from the cytosol and DcuR-P of the complex. Released DcuR-P diffuses to target promoters and binds. Uncomplexed DcuR-P in the cytosol binds to nonactivated DcuS and becomes dephosphorylated. The lower affinity between DcuR and DcuS avoids blocking of DcuS and allows rapid exchange of DcuR.

**IMPORTANCE** Complex formation of membrane-bound sensor kinases with the response regulators represents an inherent step of signaling from the membrane to the promoters on the DNA. In the C4-dicarboxylate-sensing DcuS-DcuR two-component system, complex formation is strengthened by activation (phosphorylation) *in vitro* and *in vivo*, with trapping of the response regulator DcuR at the membrane. Single-molecule tracking of DcuR in the bacterial cell demonstrates two populations of DcuR with decreased mobility in the bacteria after activation: one at the membrane, but a second in the cytosol, likely representing DNA-bound DcuR. The data suggest a model with binding of DcuR to DcuS-P for phosphorylation, and of DcuR-P to DcuS for dephosphorylation, allowing rapid adaptation of the DcuR phosphorylation state. DcuR-P is released and transferred to DNA by 3D diffusion.

## INTRODUCTION

Two-component systems (TCS) represent an important device of bacteria for sensing environmental stimuli and parameters (1, 2). The histidine sensor kinases (HK) of TCS are typically located in the cytoplasmic membrane and respond to environmental stimuli by autophosphorylation of the C-terminal transmitter or kinase domain. The latter consists of the DHp (dimerization and histidine phosphotransfer, or HisKA) domain with the consensus His residue, and the CA (catalytic and ATP-binding or HATPase_c) domain (2–4). Transfer of the phosphate group from the His residue to an Asp residue in the N-terminal receiver (REC) domain of the cognate response regulator (RR) is the basis for specific signal transfer in the TCS. In many TCS, the HK is also able to dephosphorylate the phosphorylated RR (RR-P) under nonactivating conditions to control the level of activated RR and to reset the system (1, 5). Phosphorylation of the RR results typically in improved binding to promoters and transcriptional activation of target genes.

For rapid control of the phosphorylation state of the RR by the HK, efficient interaction and phosphoryl transfer between the HK and the RR is essential. Phosphorylation requires finding and binding of the RR to HK or HK-P, whereas for dephosphorylation, complex formation between RR-P and HK is essential. This prerequisite would argue for permanent complex formation between the HK and RR proteins. However, permanent complex formation impedes the diffusion of the RR-P to the DNA and promoters, in particular when the HK is membrane-bound. Therefore, the cellular location challenges a role for stable permanent complexes in bacteria and suggests that such complexes are formed only transiently.

Studies with purified proteins suggest stable interaction of HK and RR proteins *in vivo* and *in vitro* (6–9). For *in vitro* interaction studies of membrane-integral sensors, generally preparations of the cytosolic parts of the proteins were used, which contain the kinase domain and include the sites for interaction with the response regulator. In this way, a preparation of the O_2_-sensor kinase FixL of Rhizobium meliloti, comprising the heme-binding and kinase domains forms a complex with the RR FixJ (*K_d_* ~4 μM). Presence of O_2_ inhibits the kinase activity but not FixL-FixJ complex formation (6), which indicates that the complex persists. For the complex of the osmosensor EnvZ with the RR OmpR of E. coli the situation is less clear. The cytoplasmic portion of EnvZ (EnvZc) forms a complex with OmpR (*K_d_* ~ 0.425 μM). According to Mattison and Kenney (7), phosphorylated OmpR (OmpR-P) loses the affinity for EnvZc, and binding is no longer detectable. Yoshida et al. (8) report, however, that EnvZc binds with similar affinity to OmpR and OmpR-P. The structure of a co-crystal of the cytoplasmic portion of Thermotoga maritima sensor kinase HK853 with the cognate RR RR468 reveals details of the interaction (9) of the DHp and CA domains of the HK with the REC domain of the RR. The domains interact with high specificity and recognize the phosphorylation state of the partners. Despite detailed information on the interaction and interaction sites, there is no agreement on the persistence of the interaction and its control by the phosphorylation state, both *in vitro* and in particular *in vivo* in the bacterial cells. Therefore, more detailed knowledge on the interaction of the HK and the RR, and the role of phosphorylation of either component on the interaction, is important for understanding the cooperation of the HK and RR proteins during phosphoryl transfer and dephosphorylation, and the signal transfer from the membrane to the promoters.

The DcuS-DcuR TCS of E. coli controls expression of genes for the degradation of extracellularly supplied C4-dicarboxylates (C4-DCs) such as fumarate, L-malate, L-aspartate, and succinate, under aerobic and anaerobic conditions (10–14). DcuS is composed of a periplasmic PAS (Per-ARNT-SIM) sensor domain (PAS_P_) with the site for C4-DC binding, two TM helices TM1 and TM2, a cytoplasmic signal transmitting PAS_C_ domain and a His-kinase domain (10, 15–18). Transmembrane signaling is achieved by a piston-type shift of TM2 followed by structural rearrangements to transmit the signal to the PAS_C_ domain (18–20). After stimulation by C4-CDs such as fumarate, DcuS autophosphorylates and phosphorylates DcuR, which binds with high affinity to DNA binding sites in the phosphorylated state (21–23). DcuS exists essentially as a dimer in the bacterial membrane (24). Fluorescence microscopy revealed accumulation of DcuS close to the cell poles (25, 26). The co-localization of DcuR with DcuS was independent of fumarate, which was taken as an indication that DcuS and DcuR form complexes in the phosphorylated and the nonphosphorylated states. Preliminary studies also show interaction of (full-length) DcuS with DcuR *in vivo* (27) using a bacterial two-hybrid system.

Here, interaction of DcuS with DcuR was studied with the aim to understand the role of phosphorylation and functional state on interaction, and its impact on the signal transfer in the bacterial cell. *In vitro*, interaction and complex formation was analyzed with isolated proteins of controlled phosphorylation state, and the data was compared to interaction of the proteins in the bacterial cells. Most importantly, live cell imaging could be used to track mobility changes and location of DcuR within the cells in response to activation. The data will shed light on the interaction and location of HK and RR proteins in the signal transfer by TCS from the membrane to the DNA.

## RESULTS

### Interaction of DcuS or PAS_C_-Kin with DcuR *in vivo*.

**FIG 1:**
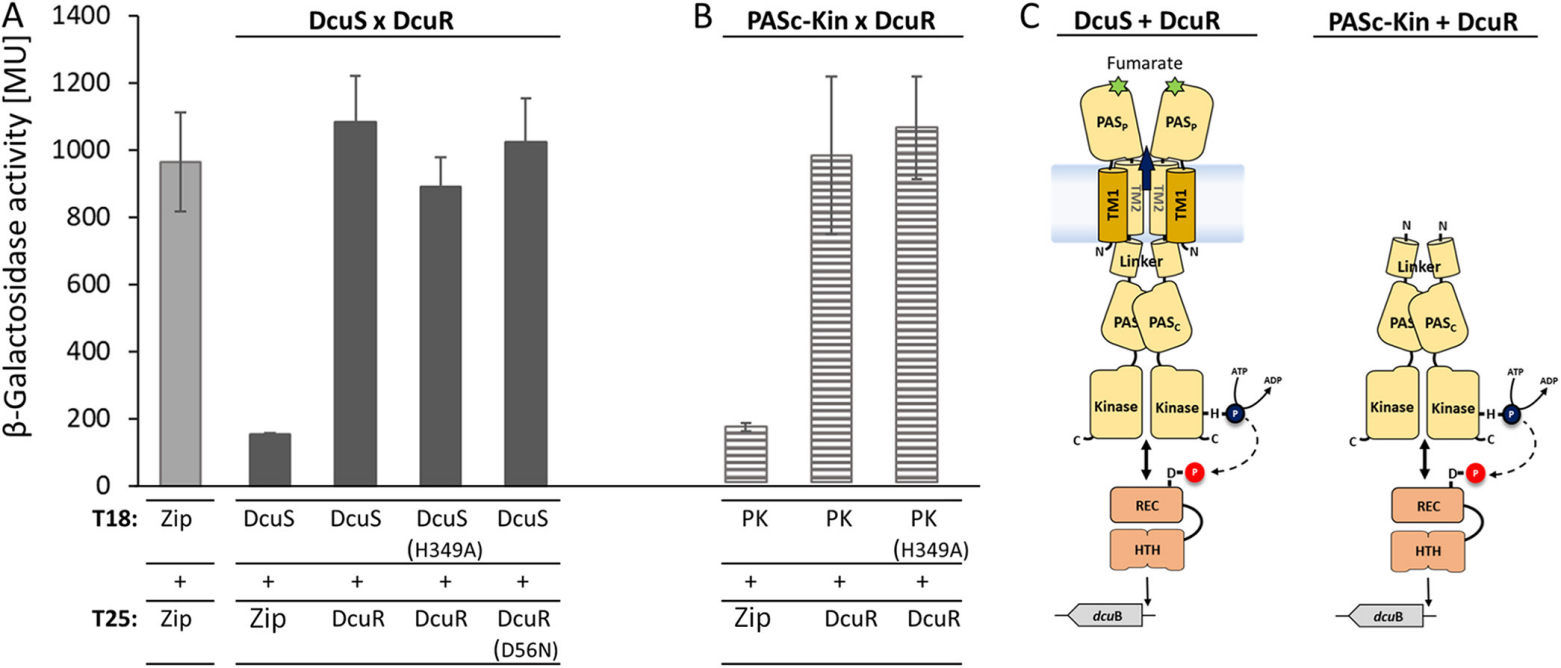
Interaction of DcuR with DcuS (A) or PAS_C_-Kin (B) *in vivo*, and schematic presentation of domains of DcuS and PAS_C_-Kin (C). Interaction was tested using the bacterial BACTH two-hybrid system. E. coli BTH101(Δ*cyaA*) was co-transformed with two plasmids that encoded proteins fused with the T25 and T18 domains of Bordetella pertussis adenylate cyclase. T25 was fused N-terminally to DcuR (T25-DcuR) and T18 C-terminally to DcuS (DcuS-T18) (Part A), or PASc-Kin (PASc-Kin-T18, or PK) (Part B). The corresponding plasmids are listed in Table 2. β-Galactosidase activities are presented in Miller units (MU). The leucine zipper pair, T18-Zip and T25-Zip, served as a positive control (28, 29), the T25-Zip/DcuS-T18, T25-Zip/PASc-Kin-T18 and T18-Zip/T25-DcuR pair served as the negative control (background β-galactosidase activity). All activities were tested in triplicates of three biological repeats. Mean values ± SD are given. Abbreviations of the domains in (C): PAS_P_, periplasmic PAS (Per-ARNT-SIM); PAS_C_, cytoplasmic PAS; TM1, TM2, transmembrane helix 1 (or 2); REC, receiver domain; HTH, helix-turn-helix DNA binding domain;SD, (standard deviation). Figure C modified from (30).

Interaction of DcuS with DcuR *in vivo* was studied using the bacterial two-hybrid system BACTH. In the BACTH assay adenylate cyclase of Bordetella pertussis is cleaved in domains T18 and T25 resulting in the inactivation of the cyclase. Fusion of the domains to interacting proteins can restore cyclase activity, which is assayed in adenylate cyclase deficient E. coli strains. Thus, recovery of cAMP production and expression of the β-galactosidase gene indicates that the test proteins interact (28, 29). Here, interaction of DcuR with the full-length DcuS (Fig. 1A) and with the truncated cytosolic variant PAS_C_-Kin of DcuS (Fig. 1B) was tested. The latter was used as an *in vivo* reference for the *in vitro* tests of PAS_C_-Kin interaction with DcuR with the isolated proteins in the following sections. The PAS_C_-Kin construct starts immediately behind TM2 and comprises the complete cytoplasmic part of DcuS with the Linker, PAS_C_ and kinase domains (Fig. 1C). In this way, it contains the domains and sites required for auto-phosphorylation, interaction with DcuR, and phosphoryl transfer to DcuR, but not the domain for fumarate sensing (13, 17). The fusion proteins retain their capacity to activate expression of the reporter genes in a fumarate dependent manner in case of DcuS and DcuR (26, 27), whereas expression of the reporter genes is fumarate independent in the case of PAS_C_-Kin and DcuR (17).

The strains with the DcuS- and DcuR-fusions showed high activity in the BACTH assay similar to the positive control that is represented by fusions of the T18 and T25 domains to the interacting Zip proteins (Fig. 1A), which agrees with earlier data (21). Mutation of the phosphorylation site in DcuS [variant DcuS(H349A)], or in DcuR [variant DcuR(D56N)] retained 94%, respectively, of wild-type activity or interaction. In the same way, inclusion of fumarate to the strain with the wild-type pair of DcuS and DcuR stimulated the interaction by less than 8%. Therefore, neither phosphorylation of the sensor kinase nor of the RR is required for strong interaction of the proteins in the BACTH assay. Strains that contained fusions of T18 or T25 to PAS_C_-Kin and DcuR, respectively, also showed high activity in the BACTH assay (Fig. 1B). Again, replacing the phosphorylation site His349 by Ala [variant PAS_C_-Kin(H349A)], did not reduce the activity (108% of the corresponding wild-type). Altogether, PAS_C_-Kin interacts strongly with DcuR *in vivo*, similar to the interaction found for DcuS with DcuR. Thus, phosphorylation of DcuS, PAS_C_-Kin or DcuR is not required for strong response in the BACTH assay.

### Effects of phosphorylation on structure and thermal stability of PAS_C_-Kin and DcuR.

Interaction of DcuS with DcuR and the effect of phosphorylation was studied *in vitro* with isolated proteins by size exclusion chromatography (SEC). In a first step, possible consequences of phosphorylation for the elution profile of the isolated compounds were investigated for proper interpretation of the profiles of the mixed samples. In fact, a shift in the elution profile can be taken as indication that the protein was phosphorylated. With the same aim, the samples were checked for changes in the thermal transition temperature upon phosphorylation.

Full-length DcuS cannot be used in SEC, since DcuS, as an integral membrane protein, must be studied either embedded in detergent micelles, or reconstituted into liposomes. In detergent, the protein loses the activity for auto-phosphorylation (21). When reconstituted in liposomes, activity is regained, but proteoliposomes are not suited for size fractionation by SEC. Therefore, the truncated soluble PAS_C_-Kin protein was used.

**FIG 2:**
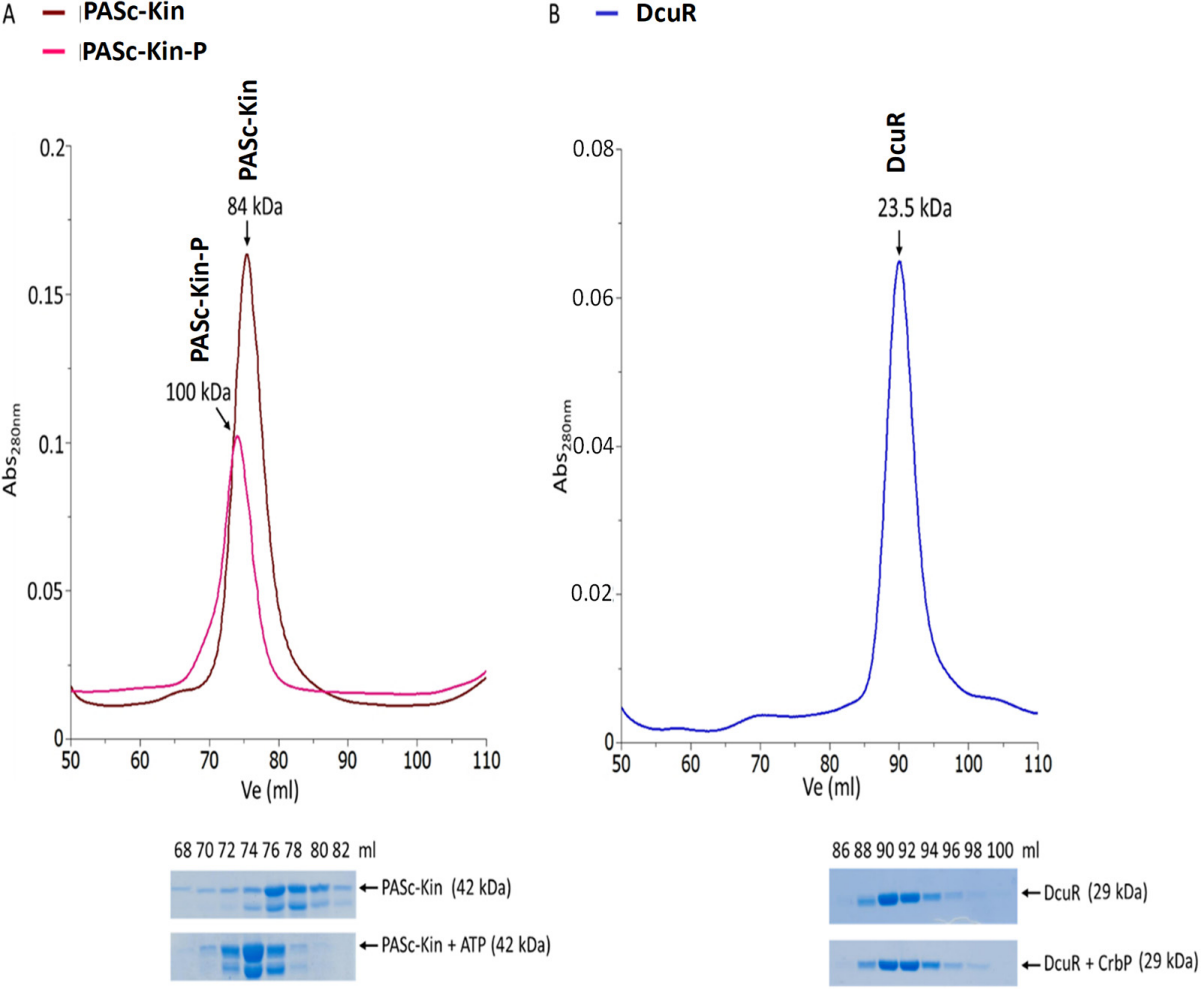
Size exclusion chromatography (SEC) of PAS_C_-Kin (A) and DcuR (B) in the phosphoryl-free and the phosphorylated states. 6His-PASc-Kinase (45 μM) and DcuR-6His (55 μM) were loaded on a HiLoad 16/600 Sephadex 200 column. For phosphorylation, the PAS_C_-Kin was incubated with ATP (1 mM) 30 min prior to SEC. DcuR was phosphorylated by incubation with 50 mM carbamoyl phosphate for 60 min. The relative molar masses (*M*_r_) of proteins (peak positions) of the SEC experiments were determined by calibration with protein standards. *SDS-PAGE*: Samples (20 μL each) from the eluted fractions were applied to SDS-PAGE and stained with Coomassie brilliant blue. Positions of PAS_C_-Kin and DcuR are indicated. Each of the experiments was performed at least in triplicate, the calibration curve for determining the *M*_r_ of the proteins by SEC is shown in Fig. S3 in the supplemental material.

Both, purified PAS_C_-Kin and DcuR eluted as single nearly symmetric peaks from a Superdex TM200 column (Fig. 2A and B). The apparent mass (*M*_r_ 84 kDa) for PAS_C_-Kin corresponded to a homodimer based on the absolute mass (40.4 kDa) of the monomer. The dimeric state of PAS_C_-Kin agrees with the dimeric state observed for full-length DcuS (18, 27, 30), the presence of major dimerization sites in the PAS_C_ and Kin domains (17, 18) and the high autophosphorylation activity of PAS_C_-Kin (14).

10.1128/msphere.00235-22.3 FIG S3 **Mass determination M_r_ of proteins by SEC by chromatography on a HiLoad 16/600 Sephadex 200 column by correlating elution volume (Ve) with the logarithm of the molar mass of proteins.** The following proteins were used for calibration: β-amylase (B-Amy) 200 kDa, BSA 66 kDa, carbonic-anhydrase (CA) 29 kDa, and aprotinin (Apr) 6.5 kDa. The relative molar masses (M_r_) of the samples PK (PAS_C_-Kin), DcuR and DcuR-P were derived from the linear fit of the calibration curve: y = A +B − xscale(X) (A= 199 ± 16 and B= −25 ± 4 scale = 10^). Download FIG S3, PDF file, 0.1 MB. Copyright © 2022 Gencheva et al. 2022 Gencheva et al. https://creativecommons.org/licenses/by/4.0/ This content is distributed under the terms of the Creative Commons Attribution 4.0 International license.

Autophosphorylation of PAS_C_-Kin by [γ^33^P]ATP is very efficient and exceeds that of reconstituted full-length DcuS by factors of 24 in extent and of 30 in rate (Fig. S1 in the supplemental material). DcuS reconstituted in proteoliposomes was phosphorylated by approximately 7% under the same conditions (21), indicating that PAS_C_-Kin is phosphorylated to high extents. The time for half-maximal labeling of PAS_C_-Kin was 1.5 min. Auto-phosphorylation of PAS_C_-Kin by ATP shifted the whole elution peak in the SEC experiment, corresponding to an increase of the *M*_r_ from 84 kDa to 100 kDa (Fig. 2A). Analysis of the peak fraction by SDS-PAGE confirmed presence of PAS_C_-Kin in the shifted peak. The increase in mass by 16 kDA is obviously much too high to just reflect the addition of the mass of two phosphate groups (0.2 kDa), suggesting that conformational changes or charge effects induced by phosphorylation cause decreased mobility and an increase of the apparent mass. The SDS-gel showed degradation products of 39 kDa, but the relative contents of the 42 and 39 kDa bands are similar in the phosphorylated and nonphosphorylated forms and thus cannot be responsible for the shift in elution volume.

10.1128/msphere.00235-22.1 FIG S1 **Autophosphorylation of DcuS and of PAS_C_-Kin.** DcuS (6 μM) was reconstituted in liposomes, and the liposomes were permeabilized by freezing and thawing before use for autophosphorylation (Janausch et al. 2002b). The permeabilized liposomes and the PAS_C_-Kinase (12 μM) were incubated in independent experiments in phosphorylation buffer with 10.2 μM [γ^3^*^3^*P]ATP. At the time points indicated samples were removed, mixed with stop solution and subjected to SDS PAGE. After electrophoresis, the radioactivity in the bands of DcuS and PAS_C_-Kin, respectively, was determined by phospho-imager and quantified after calibration (Janausch et al. 2002b). Relative radioactivity values (IOD) are presented as average values obtained from four independent experiments. *Purification and reconstitution of DcuS:* Purification and reconstitution were essentially performed as described by Janausch et al. (2002a). DcuS was purified as His_6_-DcuS encoded by pMW151. The cleared cell homogenate was centrifuged at 200.000 × *g* for 65 min at 4°C. The membrane pellet was washed twice in buffer (50 mM Tris/HCl, pH 7.7, and 3 mM EDTA) and then homogenized (1.5 mg/mL) in buffer 1 containing 2 mM dithiothreitol and the zwitterionic detergent Empigen BB (Fluka) (2%, w/v) and stirred on ice for 30-45 min. The homogenate was centrifuged (300.000 × *g* for 55 min at 4°C), and the supernatant was run by gravity through a Ni^2+^-NTA agarose column equilibrated with buffer 2 containing in addition 0,04% lauryldimethylamine oxide (LDAO). Proteins were eluted with 3 to 4 mL of buffer 2 with 100 mM imidazole and 0,04% LDAO. DcuS-His_6_ was reconstituted in liposomes of E. coli phospholipids (polar lipid extract, Avanti Polar Lipids, Alabaster, AL) as described (Janausch et al, 2002a). Protein concentration was determined using a Bradford-assay (Roth, Karlsruhe). *Phosphorylation with [γ^33^P]ATP:* The reconstituted His_6_-DcuS (6 μM) and His_6_-PASc-Kinase (12 μM) were incubated in phosphorylation buffer (50 mM TrisHCl pH7.7, 1 mM DTT, 10 mM MgCl_2_) for 45 min at room temperature. The sample with full length DcuS contained in addition sodium fumarate (20 mM). The autophosphorylation was started by the addition of 10 μM [γ^33^P]ATP (1.56 TBq/mmol). At the indicated times 10 μL sample was removed and mixed with stop solution (2x SDS loading buffer with 100 mM Tris/HCl pH6.8, 0.2% (w/v) bromophenol blue, 4% (w/v) SDS, 20% (w/v) glycerol and 200 mM dithiothreitol) and separated by SDS-PAGE. The gels were then placed on the phosphor imager plate and incubated for 16 - 19h. The autoradiogram was read with the phosphor imager and evaluated with ImageJ (Version 1.52a; Rasband, W.S., ImageJ, U. S. National Institutes of Health, Bethesda, Maryland, USA, https://imagej.nih.gov/ij/, 1997-2018.). The relative phosphorylation (%) was calculated from mean values of four independent runs. Download FIG S1, PDF file, 0.1 MB. Copyright © 2022 Gencheva et al. 2022 Gencheva et al. https://creativecommons.org/licenses/by/4.0/ This content is distributed under the terms of the Creative Commons Attribution 4.0 International license.

**FIG 3:**
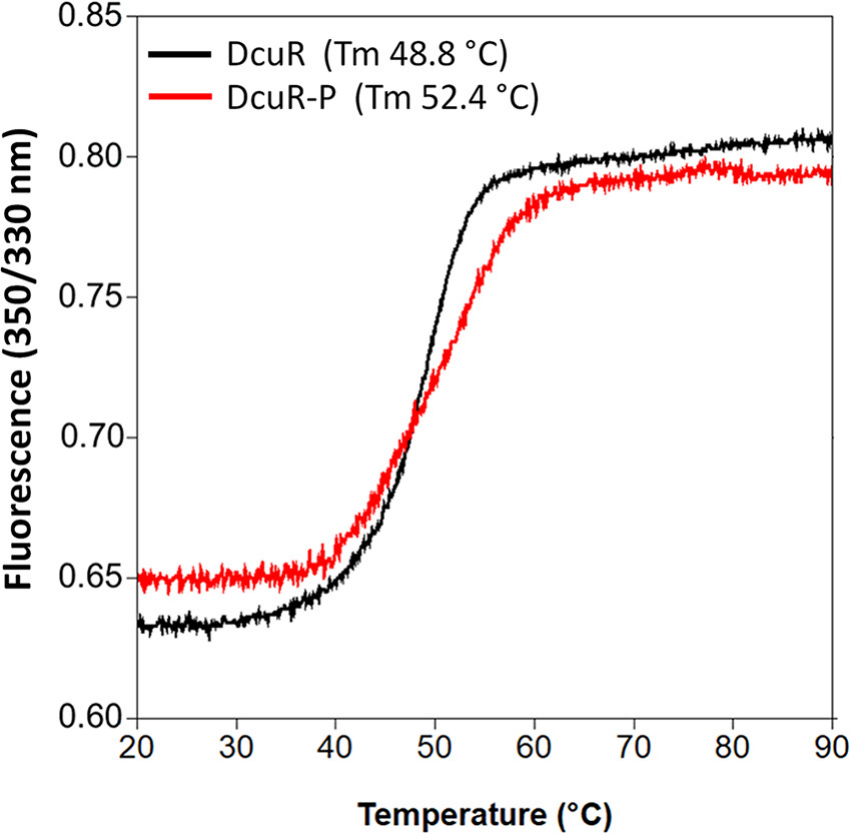
Thermal denaturation profile of DcuR in the phosphate-free (black) and phosphorylated (red) state. For thermal denaturation 10 μL samples from the corresponding SEC peaks (Fig. 2) were heated in Prometheus NT.48. The ratio of the intrinsic fluorescence recorded at 330 nm and 350 nm was used to construct the denaturation curve. The Tm values were determined from the first derivative of the curves employing the software supplied by the manufacturer. The graphs show the mean of two sets of experiments each. Thermal denaturation experiments were performed also for a set of DcuR samples which were stored at −80°C after purification and before thermal denaturation (Fig. S5 in the supplemental material). The denaturation temperature of the samples previously stored at −80°C was generally lower than that of the samples used immediately after purification (Fig. S5). However, the difference in denaturation temperature between nonphosphorylated and phosphorylated DcuR was very similar (>3°C for both sets). For comparison, the thermal denaturation profile of PAS_C_-Kin in the free (black) and phosphorylated (red) state is shown in Fig. S6.

DcuR was phosphorylated by incubation with carbamoyl-phosphate (22, 23). Phosphorylation of DcuR and formation of DcuR-P was confirmed by the gain of DNA binding capability of the protein in EMSA studies (Fig. S2 in the supplemental material). Treatment of DcuR by carbamoyl-phosphate caused a characteristic decrease in the electrophoretic mobility of the *dcuBp1* promoter DNA which exceeds that of nonphosphorylated DcuR, very similar to the behavior of DcuR-P observed earlier (22, 23). Phosphorylation had, however, no effect on the hydrodynamic properties of DcuR in the SEC experiment (Fig. 2B). DcuR and DcuR-P showed the mobility of monomeric proteins when chromatographed under the same conditions (Fig. S3). The finding contrasts an earlier study (22) that determined for DcuR-P the mass of a dimer in SEC. As an additional criterion to verify phosphorylation, the change in thermal stability of PAS_C_-Kin by phosphorylation was measured. Thermal stability of the proteins was determined from the change of tryptophan (Trp) fluorescence intensity at 350 nm and 330 nm. A change of the ratio of the intensities (I350/I330) is a measure for the shift in the emission spectrum upon unfolding (31). DcuR exhibited a characteristic transition profile upon thermal denaturation (Fig. 3), indicating that the REC and the DNA-binding domains denature coincidently with a transition temperature of 48.8°C. The transition temperature increases by 3.6°C after phosphorylation, suggesting that phosphorylation stabilizes the protein. Altogether, increased binding of DcuR-P to promoter DNA and the modified thermal stability show that DcuR is phosphorylated under the experimental conditions to large extents, even though dimerization was not visible for the DcuR-P by the SEC experiment under the present conditions. In the following experiments the role of phosphorylation of DcuR and PAS_C_-Kin on complex formation and interaction of the partners (*K_d_*) was investigated.

10.1128/msphere.00235-22.2 FIG S2 **Electrophoretic mobility shift analysis (EMSA) of DcuR and DcuR-P binding to promoter DNA of *dcu*Bp1.** EMSA was performed as described in Janausch et al. (2004). DcuR was phosphorylated by incubation with carbamoylphosphate (50 mM) for 1 h. DcuR-P (6 μM) and DcuR (6 μM) were mixed with *dcuB*p1 promoter DNA (2.5 nm) and subjected to EMSA. Lane (1) contains *dcuB*p1, lane (2) *dcuB*p1and DcuR-P, lane (3) *dcuB*p1 and DcuR, lane (4) DNA size markers (labelled in Bp). The DNA bands corresponding to *dcuB*p1, and complexes C1 and C2 of *dcuB*p1 with DcuR and DcuR-P, respectively, are indicated by arrows. Download FIG S2, PDF file, 0.1 MB. Copyright © 2022 Gencheva et al. 2022 Gencheva et al. https://creativecommons.org/licenses/by/4.0/ This content is distributed under the terms of the Creative Commons Attribution 4.0 International license.

10.1128/msphere.00235-22.5 FIG S5 **Thermal transition temperature (Tm) for the denaturation of DcuR and phospho-DcuR (DcuR-P)**. Average values were calculated from Tm values obtained from DcuR measured direct after purification and after storage at – 80°C; the data from individual experiments are presented in the Table. Download FIG S5, PDF file, 0.1 MB. Copyright © 2022 Gencheva et al. 2022 Gencheva et al. https://creativecommons.org/licenses/by/4.0/ This content is distributed under the terms of the Creative Commons Attribution 4.0 International license.

10.1128/msphere.00235-22.6 FIG S6 **Thermal denaturation profile of PAS_C_-Kin in the free (black) and phosphorylated (red) state.** Thermal denaturation was performed in duplicate for each of two samples. One set of data acquisition was performed with proteins, which where thermally denatured directly after purification. The other data set acquisition was performed with proteins that were previously stored at –80°C. Download FIG S6, PDF file, 0.1 MB. Copyright © 2022 Gencheva et al. 2022 Gencheva et al. https://creativecommons.org/licenses/by/4.0/ This content is distributed under the terms of the Creative Commons Attribution 4.0 International license.

### Complex formation between PAS_C_-Kin and DcuR: The role of phosphorylation.

**FIG 4:**
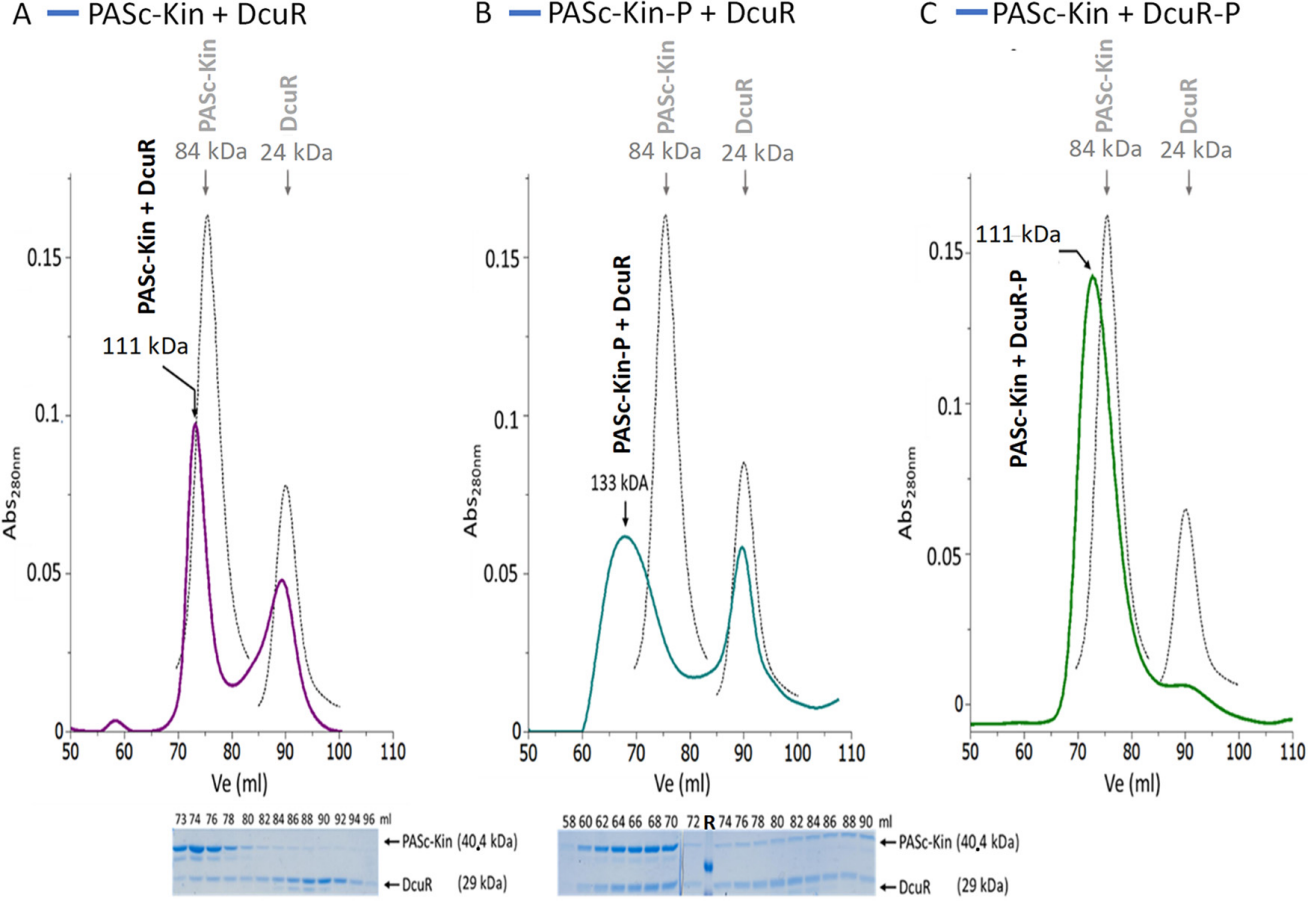
SEC of mixtures of PAS_C_-Kin and DcuR (phosphate-free or phosphorylated) in various combinations: (A) PAS_C_-Kin × DcuR, (B) PAS_C_-Kin-*P* × DcuR, and (C) PAS_C_-Kin × DcuR-P. PAS_C_-Kin and DcuR were mixed in a molar ratio of 1:1.44 (A), 1:2 (B) and 1:1.4 (C) with PAS_C_-Kin at a concentration of 40.4 μM. Phosphorylation of PAS_C_-Kin and DcuR, respectively, was performed as described in Fig. 2 before mixing the samples. SDS-PAGE (parts A and B) and SEC were performed as for Fig. 2. The protein elution profiles were recorded, and the contents of PAS_C_-Kin and DcuR in the peak fractions of part (A) and (B) were analyzed by SDS-PAGE (lower part of the figure). The elution profiles (black lines) are compared to the elution profiles of the proteins chromatographed individually (light gray lines) as in the experiment of Fig. 2. The SDS-PAGEs were used for quantification of the bands by integration of the area and intensity of the stain in the SDS-PAGE by ImageJ. The relative molar contents of the proteins and their molar ratio (see main text) were calculated from the protein staining intensity and the molar masses (18). Experiments were performed at least in triplicate, and the molar contents and ratios are the means from three independent experiments (±SD). The lane labeled ‘R’ in the SDS-PAGE of (B) shows size markers of 42 and 29 kDa.

Formation of complexes between PAS_C_-Kin and DcuR was analyzed by co-incubation of the proteins followed by SEC, which allows differentiation of independently or jointly eluting proteins (Fig. 4). The proteins were incubated with DcuR in slight excess to PAS_C_-Kin to enable full saturation of PAS_C_-Kin, and the proteins were mixed in different phosphorylation states. A shift of the chromatogram toward lower volumes was taken as indication for formation of a complex. Since the elution volume might not correspond to the actual molecular weight, the stoichiometry was estimated additionally from the amount of the two proteins observed in SDS-PAGE of the corresponding fractions.

When unphosphorylated proteins were co-incubated (PAS_C_-Kin and DcuR), in the SEC experiment the peak of free PAS_C_-Kin at 84 kDa was replaced by a major peak that eluted at a volume corresponding to a protein of 111 kDa (Fig. 4A). The slightly asymmetric peak was followed by a peak with a position nearly identical to that of free DcuR (*M*_r_ 24 kDa), containing the fraction of freely migrating DcuR. SDS-PAGE analysis of the 111 kDa peak revealed co-migration of DcuR with PAS_C_-Kin suggesting that the mass of 111 kDa is a result of PAS_C_-Kin × DcuR interaction. The peak fractions contained approximately 0.11 mol DcuR/mol PAS_C_-Kin, as concluded from SDS-PAGE and quantitative evaluation of the protein content (Fig. 4A, SDS-PAGE), whereas the major portion of DcuR was found in the fractions of the 24 kDa peak. The sub-stoichiometric levels of DcuR in the PAS_C_-Kin peak and the tailing of the peaks indicate dissociation of a labile PAS_C_-Kin × DcuR complex (see below, Fig. 6) during chromatography, and the 111 kDa peak is probably an overlay of complexed and uncomplexed PAS_C_-Kin. There is no indication for another form of modification of PAS_C_-Kin by DcuR.

When PAS_C_-Kin was first incubated with ATP to produce phosphorylated PAS_C_-Kin (PAS_C_-Kin-P) and then with DcuR, PAS_C_-Kin eluted in a broad peak with an apparent mass of 133 kDa at the maximum (Fig. 4B). Analysis of the SDS-PAGE indicates that the corresponding fraction contained 0.86 ± 0.3 mol DcuR/mol PAS_C_-Kin (Fig. 4B). The data suggests a 1:1 stoichiometry of PAS_C_-Kin and DcuR, and a 2:2 complex because PAS_C_-Kin is present as a dimer. This is supported by the mass of the complex deduced from the elution volume (*M*_r_ 133 kDa), which corresponds approximately to the combined calculated absolute masses of (dimeric) PAS_C_-Kin-P (80.8 kDa) and dimeric DcuR (58 kDa). Therefore, the contents of PAS_C_-Kin and DcuR in the SDS-PAGE and the mass of the complex in the SEC experiment suggest a hetero-tetramer from (PAS_C_-Kin-P)_2_ and DcuR_2_. We denote the complex as [PAS_C_-Kin × DcuR]_2_ × P_2_, as the location of the phosphate group (at the phosphorylation site in the DHp domain [His349], or in DcuR [Asp56]) in the final complex is not known. It is, however, unlikely that the complex represents a (PAS_C_-Kin × DcuR-P)_2_ complex, since the corresponding complex in Fig. 4C shows a different mobility (111 kDa). The complex is of sufficient stability to survive the SEC, and excess DcuR eluted in a further peak with the mobility of free DcuR (*M*_r_ 24 kDa) as in the other experiments.

In a third experiment, DcuR was phosphorylated first by incubation with carbamoyl-phosphate and mixed then with PAS_C_-Kin and subjected to SEC. There was only one main protein peak eluting at a volume corresponding to an apparent *M*_r_ of 111 kDa (Fig. 4C). Thus, at first glance the result is similar to the case where both proteins are unphosphorylated (Fig. 4A). However, phosphorylation obviously strongly strengthens complex formation, since despite loading similar amounts (1:1.4 and 1:1.44) onto the column, only low amounts of free DcuR-P (*M*_r_ 24 kDa) were found, in strong contrast to the chromatogram of PAS_C_-Kin with DcuR (Fig. 4A). Appearance of the *M*_r_ 111 kDa peak, together with the disappearance of the *M*_r_ 84 and *M*_r_ 24 kDa peaks of free PAS_C_-Kin and DcuR-P indicates the formation of a stable PAS_C_-Kin × DcuR-P complex. Again, considering that PAS_C_-Kin is a dimer, it can be speculated that the complex has a 2:2 composition.

Altogether, the experiments show that PAS_C_-Kin forms complexes with DcuR, which differ in their hydrodynamic properties depending on the protein phosphorylation. The complexes are stable in the SEC experiments when one of the proteins was in the phosphorylated state, indicating that the dissociation rate of the complexes is small compared to the time scale of the separation process (>30 min). Remarkably, the apparent masses of the complexes are different when phosphorylation was performed by ATP at PAS_C_-Kin or by carbamoyl-phosphate at DcuR, indicating structural differences that hamper accurate determination of subunit stoichiometries.

**FIG 5:**
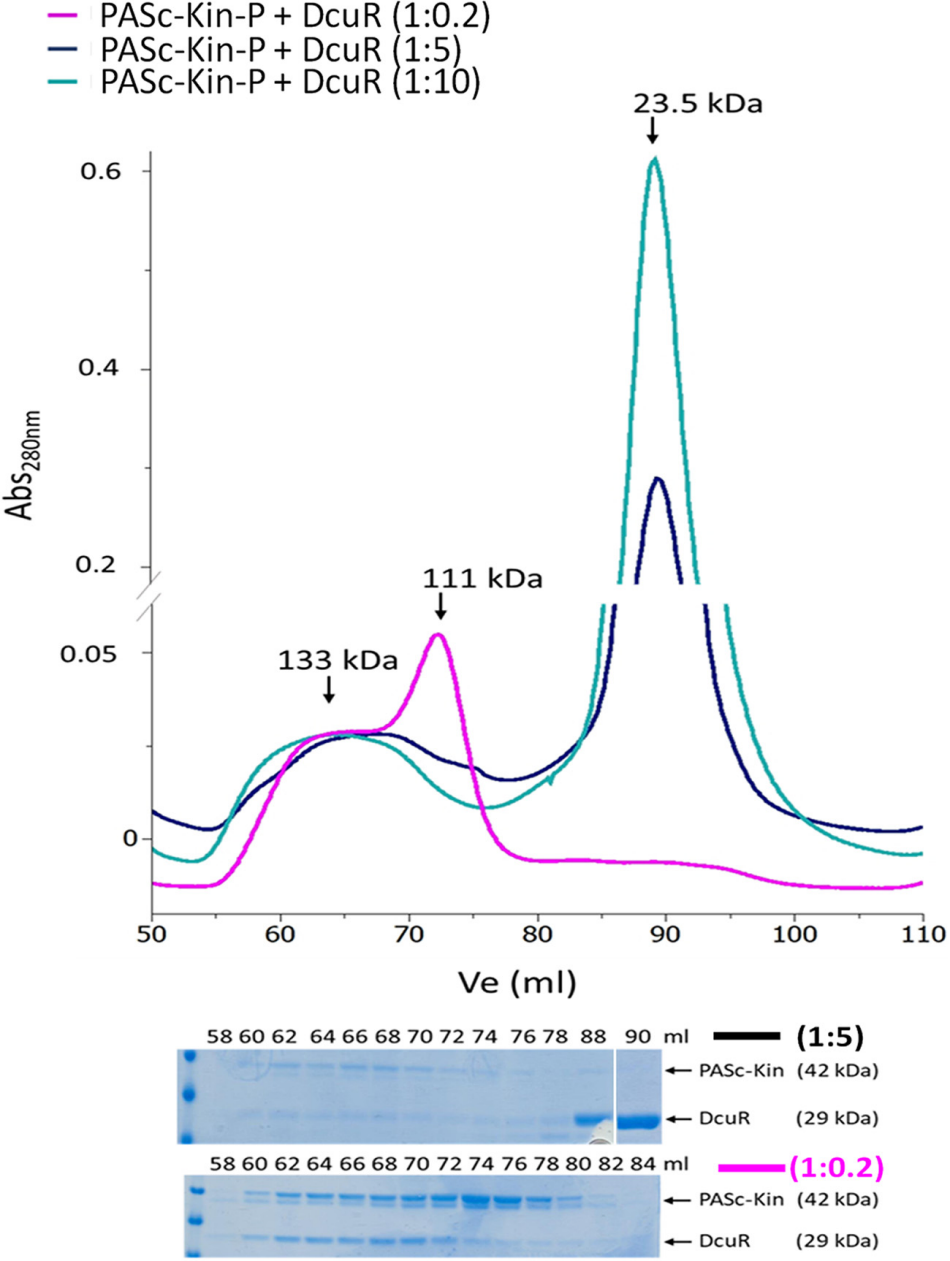
Complex formation of PAS_C_-Kin-P and DcuR at different molar ratios. PAS_C_-Kin was phosphorylated by incubation with ATP, mixed with DcuR and subjected after 10 min to SEC as described for Fig. 4. The concentrations of PAS_C_-Kin-P were 110 μM, 22 μM and 22 μM, respectively for the 1:0.2, 1:5 and 1:10 mixtures (DcuR was added at 22 μM, 110 μM, and 220 μM concentrations respectively). SEC, SDS-PAGE and quantitative evaluation of SDS-PAGE for the contents of PAS_C_-Kin and DcuR were performed in duplicate, other details as described for Fig. 4.

To find out whether also other types of complexes can be formed, interaction of PAS_C_-Kin-P and DcuR was investigated at different molar ratios in the initial mixture (Fig. 5). When PAS_C_-Kin-P was mixed with sub-stoichiometric levels of DcuR (molar ratios 1:0.2), the SEC elution profile showed two peaks, representing complexes with an apparent mass of *M*_r_ 133 kDa and 111 kDa, respectively. Again, the 133 kDa fraction corresponds to the 2:2 PAS_C_-Kin-*P* × DcuR complex, whereas according to the SDS-PAGE pattern the 111 kDa peak seems to contain unbound PAS_C_-Kin-P. There was no peak corresponding to free DcuR, indicating that all DcuR molecules are bound to PAS_C_-Kin. With higher levels of DcuR in the initial mixture, the *M*_r_ 111 kDa peak vanished; at the same time, a peak corresponding to free DcuR (*M*_r_ 23.5 kDa) appeared in the SEC elution profile. Thus, it seems that only one type of complex is formed from PASc-Kin-P and DcuR, and that the 133 kDa peak is a product of both proteins.

The SEC experiments showed that phosphorylation of DcuR or PASc-Kin strengthens complex formation. For more quantitative information, binding constants were determined employing Microscale thermophoresis (MST). This method uses the changes in the thermodiffusion coefficient of (fluorescently labeled) proteins due to the differences in size, charge and/or solvation energy upon interaction with other molecules or proteins (32, 33). Diffusion is induced by a microscopic temperature gradient without imposing significant mechanical or shear forces (32, 34). The change in fluorescence intensity in the heated area of the capillary at steady state conditions is proportional to the change in local concentrations of the labeled proteins due to interaction with a binding partner, and the corresponding change in the thermo-diffusion coefficient. For the experiments, DcuR was fluorescently labeled by coupling it genetically to enhanced yellow fluorescent protein (eYFP) by a short linker, yielding a construct still active *in vivo* in gene regulation (26).

**FIG 6:**
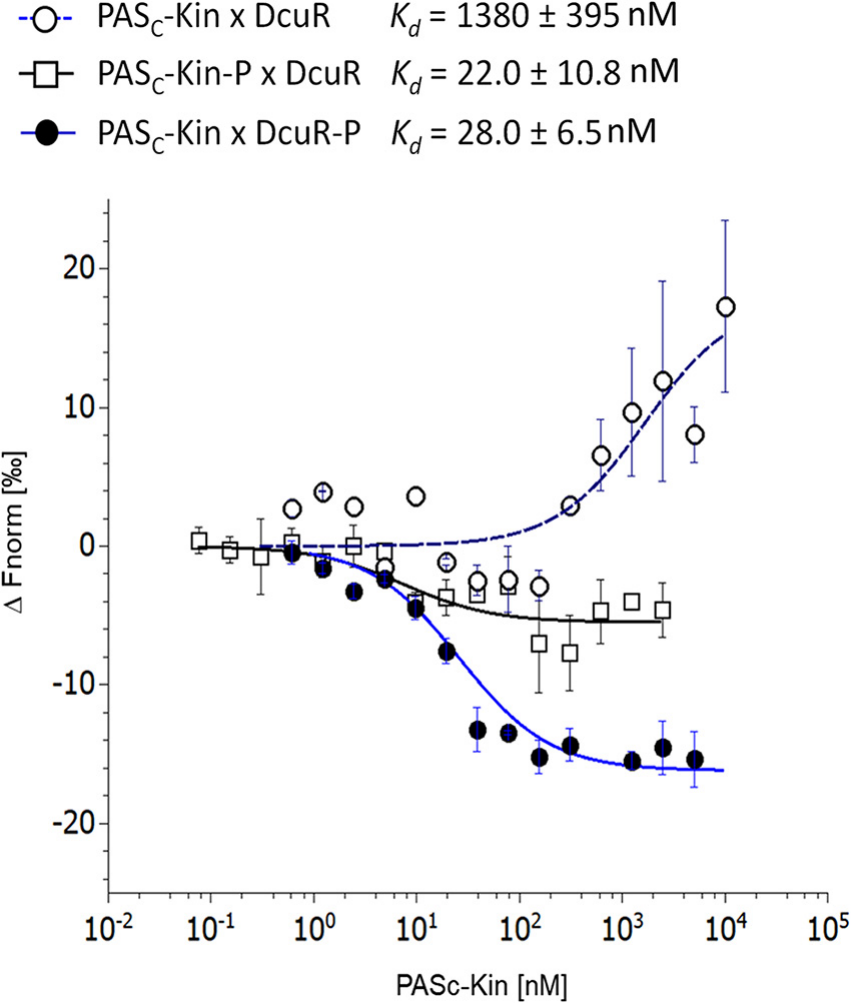
Quantitative evaluation of PAS_C_-Kin and DcuR interaction by microscale thermophoresis PAS_C_-Kin × DcuR (-○-), PAS_C_-Kin-P × DcuR (-□-) and PAS_C_-Kin × DcuR-P (-●-). eYFP-L-DcuR was applied at 0.1 μM, and His_6_-PAS_C_-Kin in concentrations from 10^−1^ to 2 × 10^4^ nM as indicated. PAS_C_-Kin and eYFP-DcuR were phosphorylated by ATP and carbamoyl-phosphate, respectively, as described in Fig. 2. Fluorescence difference (ΔFnorm) per thousand [‰] is shown for eYFP-L-DcuR (open circles, left Y-axis) and eYFP-DcuR~P (closed circles, right Y-axis). The lines correspond to a fit based on a 1:1 binding model. The errors present the uncertainty of the fitted *Kd* value as given by the fitting routine.

In the MST experiment, the diffusion of eYFP-L-DcuR was monitored at different PAS_C_-Kin concentrations (Fig. 6). The resulting binding curves can be reasonably well fitted with a function based on a simple 1:1 binding mechanism in all three cases, in agreement with a 1:1 stoichiometry in the complex and no distinguishable cooperative interactions. The *K_d_* values for complex formation between PAS_C_-Kin and eYFP-L-DcuR refer to monomer concentrations and were 1384 ± 395 nM for de-phosphorylated components and decreased to 22 ± 11 and 28 ± 7 nM, respectively, when PAS_C_-Kin-P or eYFP-L-DcuR-P was used in its phosphorylated form. If dimer concentrations are used for the model, corresponding to a complex of dimers (2:2), the corresponding *K_d_* values have half the value, but the curves are identical (not shown), and the concentration of complex formed can be calculated either way. Thus, at the concentrations employed in the SEC experiments the maximally possible amounts of complexes should be formed in all three cases. However, a lower affinity is often accompanied by an increased dissociation rate of the complex, explaining the low amount of complex detected in the SEC elution profile when the two unphosphorylated proteins were initially mixed but separated during the SEC run (see Fig. 2 and 4). Furthermore, the affinity of the two proteins to each other is very similar if one of the binding partners is phosphorylated, no matter which one it is.

### Cellular levels of DcuS and DcuR.

Assessing the role of DcuS and DcuR and their complexes in bacteria requires information on the absolute quantitative levels of the proteins in the bacteria grown with and without fumarate. Previously, the absolute DcuS levels of isolated membranes of E. coli were determined by SRM mass spectrometry with spiking-in of known amounts of ^13^C-labeled proteotypic peptides of DcuS (35). This approach was necessary to enable quantification of DcuS in E. coli due to the very low number of molecules per cell, both with and without fumarate present in the medium (Table 2, Table S1 in the supplemental material). To provide additional information on absolute levels of the more abundant DcuR we used global protein profiling of E. coli cell lysates upon aerobic and anaerobic growth in the presence or absence of fumarate to determine intensity based absolute quantitative (iBAQ) values. iBAQ values can be used to calculate the number of molecules per cell (36). DcuR was identified and quantified by six peptides in the current analysis. During aerobic growth 197 pmol mg^−1^ of protein DcuR were measured (Table 2, Table S1). The levels increased when cells were grown in the presence of fumarate (830 pmol mg^−1^) or upon anaerobic growth (without fumarate 629 pmol mg^−1^; with fumarate 979 pmol mg^−1^). The contents correspond to 50 and 56 molecules of DcuR per cell for aerobic and anaerobic growth with fumarate.

10.1128/msphere.00235-22.7 TABLE S1 Estimation of DcuR levels from iBAQ values estimated in a global profiling approach. Download Table S1, DOCX file, 0.02 MB. Copyright © 2022 Gencheva et al. 2022 Gencheva et al. https://creativecommons.org/licenses/by/4.0/ This content is distributed under the terms of the Creative Commons Attribution 4.0 International license.

### Single-molecule tracking (SMT) of DcuR reveals accumulation at the cell poles and strongly reduced mobility throughout the cells after activation of DcuS.

**FIG 7:**
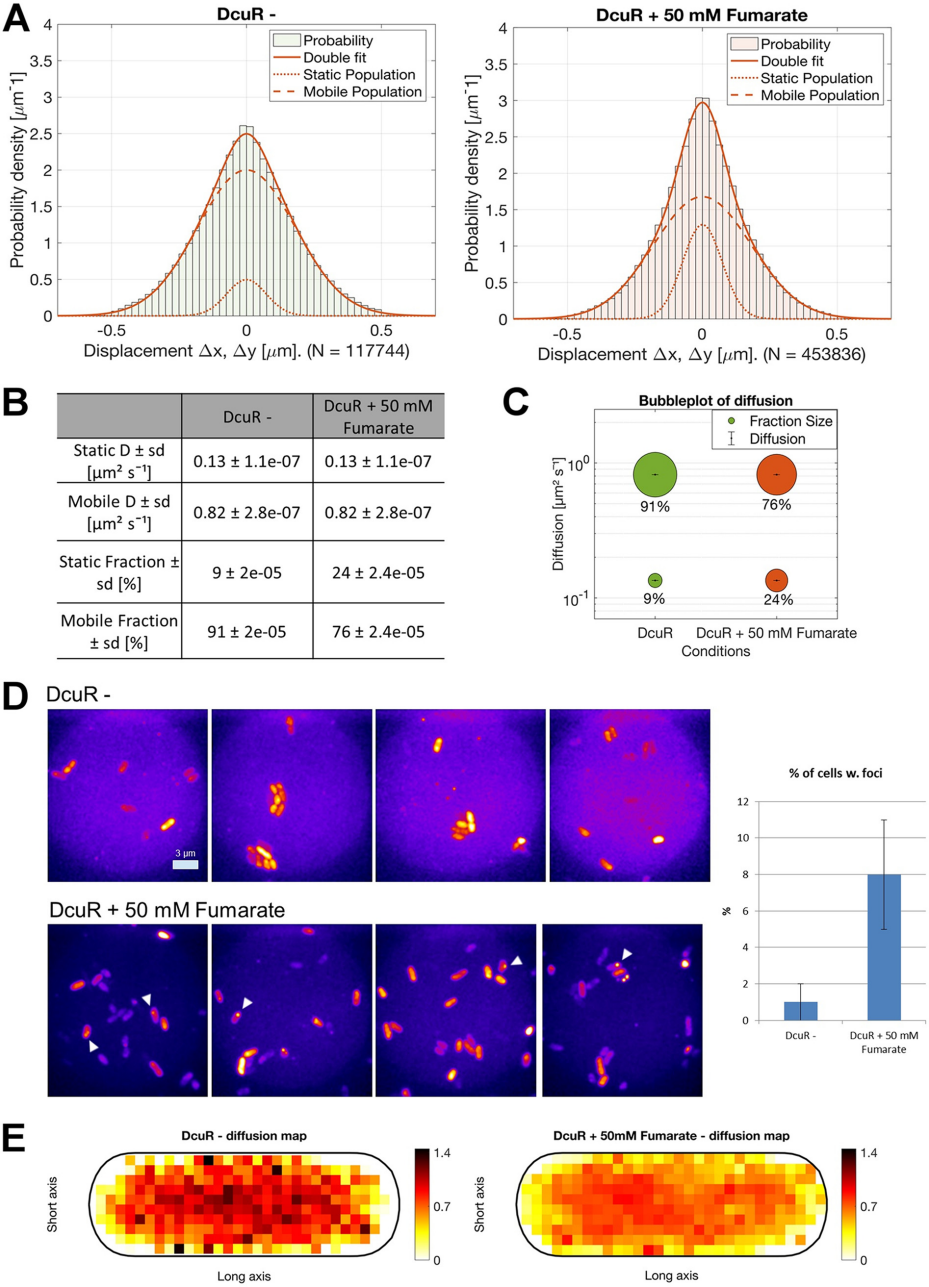
YFP-DcuR single-molecule dynamics. (A) Two-population fit of YFP-DcuR single molecule dynamics, using simultaneous Gaussian-mixture-modeling (GMM) under normal growth conditions in M9 minimal-media (−) and with added 50 mM fumarate (+). (B) Table of calculated diffusion values and population sizes, (C) bubble-plot illustrating the relative fraction sizes and average diffusion constants (D [μm^2^s^−1^]) of the populations. (D) Summation of tracked fluorescence signals for YFP-DcuR under normal growth conditions in M9 minimal-media (−) and with added 50 mM fumarate (+) and percentage of cells showing localized fluorescence foci (*n* = 150 cells per replicate) examples marked with white arrow; scale bar 3 μm. (E) Heat maps showing the distribution of diffusion of YFP-DcuR (dark red: higher diffusion, white: lower diffusion) normalized in an average-sized cell in M9 minimal-media (−) and with added 50 mM fumarate (+).

DcuS forms clusters at the cell membrane, predominantly at the cell poles (25, 26), that recruit additional signaling components. In contrast to this, DcuR was found to be evenly distributed throughout the cells, even after induction of signaling via DcuS (26). To test the idea that DcuR becomes more tightly engaged in binding to DcuS after stimulation of the kinase, we employed SMT, which not only greatly increases temporal but also spatial resolution of fluorescently labeled molecules. We used an N-terminal YFP fusion to DcuR that was shown to fully complement for the function of DcuR in an earlier study (26). The fusion yielded well visible single-molecule point spread functions, which were automatically tracked using program utrack (37). We analyzed trajectories of 5 steps or more using Gaussian Mixture Modeling (GMM), which plots the probability of different step lengths within a coordinate system (38). Fig. 7A shows that two Gaussian distributions, one for slow-molecules (dotted line) and one for fast molecules (dashed line), were required to adequately fit the observed step size distribution; the solid line indicates the result of the two-population fit. Therefore, DcuR appears to move as two populations, one corresponding to freely diffusing molecules (highmobile fraction, diffusion constant D = 0.82 μm/s, 91% of the molecules), and one with D = 0.13 μm/s (Fig. 7B, note that SD values are fitting errors) that is bound to a much larger structure, most likely to DNA. After addition of 50 mM fumarate, which will saturate the system, mean mobility of DcuR (or DcuR-P, respectively) strongly decreased, best seen by the much narrower distribution around “0” in the GMM plot in Fig. 7A. Low mobility was caused by a strong increase in the low-mobile population to 24%, indicated by bubble size in Fig. 7C, at the expense of the high-mobile population. Interestingly, stacking of image planes from the movies revealed the existence of fluorescent, membrane-located *foci* for YFP-DcuR, mostly at the cell poles – their number increased from 1% to 8% of cells (Fig. 7D). Of note, because of higher signal to noise ratio and spatial resolution (less than 50 nm) in SMT experiments, these foci were undetectable using epifluorescence microscopy. A straight forward explanation for the foci is YFP-DcuR transiently accumulating at DcuS clusters, which also show highest inclination toward the cell poles (26).

To analyze if reduced mobility of DcuR-P after stimulation of DcuS occurs also at other subcellular locations, we generated speed maps, in which average diffusion constants of molecules are plotted using a 100 nm grid. From Fig. 7E, it can be clearly seen that average diffusion constants strongly decreased in the central part of the cell containing the nucleoids, but also at the cell membrane. Because phosphorylation of DcuR *per se* will not alter its diffusion constant, which scales with the radius of molecules, and a possible dimerization would not lead to a strong increase seen for the low-mobile molecules, reduced mobility on the nucleoids can be explained by stronger engagement in promoter binding, while reduced mobility at the cell membrane likely reflects longer dwelling at receptor complexes.

## DISCUSSION

### DcuS exists in complex with DcuR: Role for rapid adaptation of DcuR phosphorylation state.

PAS_C_-Kin forms complexes with DcuR in particular when one of the proteins is in the phosphorylated state. The sites for the interaction of sensor kinases with response regulators are located in the kinase domain (9, 39, 40), which is present in PAS_C_-Kin together with the adjacent PAS_C_ domain. The *in vitro K_d_* values for the phosphorylated proteins are in the nM range which is characteristic for stable protein complexes (41, 42). The unphosphorylated proteins are characterized by weaker complex formation (*K_d_* = 1380 ± 395 nM) and probably decreased kinetic stability, namely, an increased off-rate. The *K_d_* values that were determined for interaction of DcuR with PAS_C_-Kin might be different for interaction with DcuS. The BACTH data confirm, however, that the DcuS × DcuR and PAS_C_-Kin × DcuR pairs interact *in vivo* as well, both in the fumarate activated and the inactive state.

**FIG 8:**
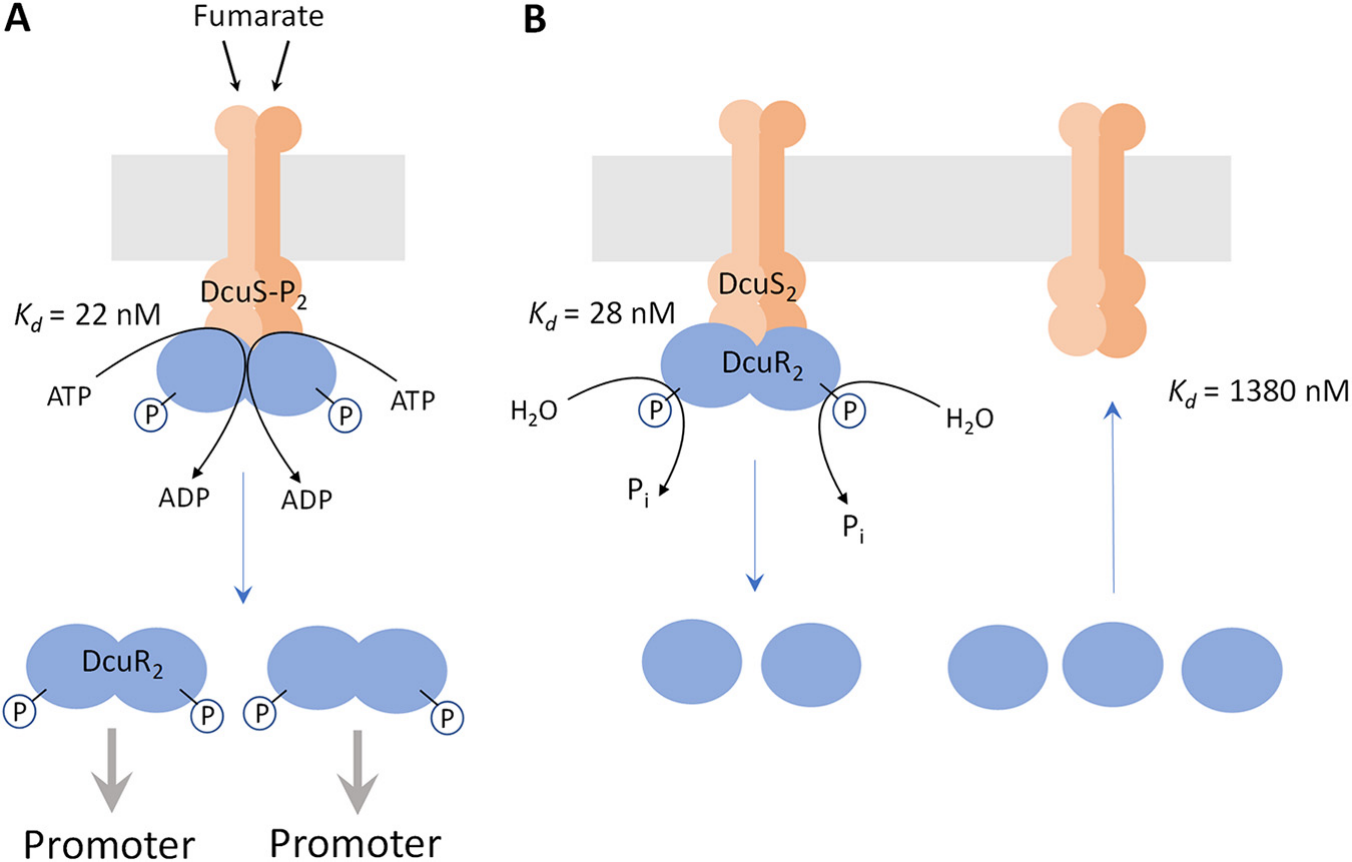
A model for the presence of DcuS and DcuR in bacteria in the presence (A) and absence (B) of fumarate: DcuS × DcuR complex formation and its role doe DcuR phosphorylation or DcuR-P dephosphorylation. The levels of DcuR, DcuS, and of the DcuS × DcuR complexes are given in Table 1, and the number of the respective proteins in the scheme is not equivalent to their level. Generally, levels of DcuR exceed those of DcuS by factors of 29.4 or higher (Table 1). (A) In the presence of fumarate, DcuS_2_ is activated; (DcuS-P)_2_ binds DcuR_2_ with high affinity (*K_d_* 22 nM) and (DcuR-P)_2_ is produced. A stable DcuS_2_ × (DcuR-P)_2_ complex is formed, without free DcuS_2_ left (see Table 1). Ongoing activation of DcuS (presence of fumarate) stimulates release of (DcuR-P)_2_ which finds DNA and promoters by 3D-diffusion. (B) In the absence of fumarate, DcuS is not phosphorylated and binds (DcuR-P)_2_ with high affinity (*K_d_* 28 nM). The intrinsic phosphatase of DcuS dephosphorylates (DcuR-P)_2_, resulting in the release of DcuR (*K_d_* 1380 nM). Cytosolic DcuR is assumed to exist as apo-DcuR in the phosphate-free state (presented as monomer) or phosphorylated (DcuR-P)_2_.

The complex formation between DcuS and DcuR has major implications on the cellular organization of the proteins (Fig. 8). The contents of DcuR exceed those of DcuS under all conditions (Table 1). Complex formation in the bacteria can be estimated by the law of mass action from the *K_d_* values of the complexes and the concentrations of DcuS and DcuR in the bacteria. For this estimation averaged cellular concentrations of DcuS and DcuR and the *K_d_* values of the PAS_C_-Kin and DcuR proteins are used. With these parameters, the concentrations of the DcuS × DcuR-P complex (when the system is activated by fumarate) approach those of total DcuS (Table 1), meaning that DcuS exists completely in the complexed form. In the absence of fumarate when DcuS and DcuR are not phosphorylated, still most of the DcuS is complexed during aerobic and anaerobic growth (87% and 96%, respectively).

**TABLE 1:** DcuR and DcuS levels at different growth conditions in E. coli W3110

	DcuR		DcuS		DcuS × DcuR
Growth	pmol mg^−1^	μM*	pmol mg^−1^	μM*	μM*
Aerobic	197	9.9	6.7	0.34	0.297
+Fumarate (20 mM)	830	41.5	7.5	0.38	0.38
Anaerobic	629	31.5	6.7	0.34	0.326
+Fumarate (50 mM)	979	49.0	5.8	0.29	0.29

Average values from three (DcuS) or four (DcuR) biological replicates are presented. The concentration of the Dcus × DcuR complex (μM*) is calculated from law of mass action for complex formation from DcuS (μM*) and DcuR (μM*) and the corresponding *K_d_* values (Fig. 6). For details, see Materials and Methods.

Complex formation as described here was not seen in an earlier study on the cellular localization of the proteins using epifluorescence microscopy (26). Static motion of some molecules is blurred out by epifluorescence, but can be accurately visualized by single-molecule tracking (SMT). By employing SMT, we find here that a low but considerable number of cells shows inducible polar accumulation of DcuR-P after addition of fumarate. The microscopy data support increased DcuS-DcuR-P complex formation after activation which is in line with the *in vitro* situation. The *in vitro* complexes of PAS_C_-Kin with DcuR are apparently more stable or permanent than the DcuS – DcuR complexes. *In vivo*, DcuR-P stays in complex with DcuS for at least several seconds. We show that after stimulation of DcuS, DcuR (i.e., DcuR-P) markedly slows down and its low-mobile fraction increases strongly, at the expense of freely diffusing molecules also in the vicinity of the cell membrane. At the membrane, reduced diffusion constants of DcuR likely represent binding to DcuS.

DcuS is a member of the HisKA family of kinases and carries an E_350_xxN_353_ phosphatase motif (43) in the DHp domain (Fig. S4 in the supplemental material). Therefore, DcuS is supposed to also harbor phosphatase activity that comes into play in the absence of C4-DCs when surplus DcuR-P has to be dephosphorylated for reestablishing the ground state (Fig. 8). The studies show strong complex formation and low *K_d_* value also for this situation when DcuS and DcuR-P are present, to allow DcuS × DcuR-P complexes to catalyze DcuR-P dephosphorylation.

10.1128/msphere.00235-22.4 FIG S4 **Amino acid sequence motif with the E/DxxN/T phosphatase motif in sensor kinases DcuS and KdpD of the HisKA kinase subfamily, and comparison to the DxxxH/Q phosphatase motif of sensor kinase NarX, a sensor kinase of the HisKA_3 subfamily.** The phosphatase motif is located in helix α1 of the DHp domain. The sequence is located next to the conserved His residue that is phosphorylated (H349 in DcuS). Mutation of E(350) results in inactivation of DcuS function, whereas mutation of N(353) renders DcuS to a partial ON phenotype which is compatible with inactivation of a phosphatase motif. The numbers above the sequence of DcuS and below that of NarX are related to the positions of the corresponding residues in the proteins. Figure modified from (Huynh et al. 2010). Download FIG S4, PDF file, 0.1 MB. Copyright © 2022 Gencheva et al. 2022 Gencheva et al. https://creativecommons.org/licenses/by/4.0/ This content is distributed under the terms of the Creative Commons Attribution 4.0 International license.

Overall, DcuS is present in E. coli essentially in the complexed state that is preferentially formed when DcuS-P and DcuR, or DcuS and DcuR-P, respectively, are present. Tight complexes enable rapid adaptation of the DcuR phosphorylation state in either direction for efficient regulation of transcription by the DcuS-DcuR TCS.

### Release of DcuR from the membrane-bound DcuS-DcuR complex for DNA search and binding.

For DNA binding and transcriptional regulation, DcuR-P has to dissociate from the membrane-located DcuS × DcuR-P complex. The number of DcuR molecules exceeds that of DcuS by a factor of 29 or more (Table 1). Therefore, most of the DcuR is cytosolic, both in absence and presence of fumarate, but distributed between free DcuR and a DNA-bound fraction. In the SMT experiments, following stimulation of DcuS, DcuR (i.e., DcuR-P) markedly slows down and its low-mobile fraction increases strongly, at the expense of freely diffusing molecules. Reduced diffusion was observed within the center of the cells, likely representing increased binding of DcuR-P to promoters on the chromosome(s). While *in vitro* the dissociation of PAS_C_-Kin × DcuR-P is slow, this seems to be different according to the STM data for DcuS × DcuR-P *in vivo*, when the complex is formed with full-length DcuS. The truncated PAS_C_-Kin is not able to adapt all functional states of DcuS (17), and DcuS requires also the C4-DC transporters DctA or DcuB as co-regulators for the control of activity (13, 44–46). Therefore, the DcuS-DcuR complexes, which are mostly located in the membrane close to the cell pole, obviously represent the site for rapid adaptation of the DcuR phosphorylation state. The genes *dcuB*, *dctA* and *frdABCD* that are directly regulated by DcuR (10, 11, 47–49) have differing chromosomal locations. In addition, few more genes have been suggested for direct regulation by DcuS-DcuR (47, 50). This situation is hardly compatible with regulation by a membrane-bound DcuS-DcuR complex, unless all gene loci translocate to the cell poles, which seems unlikely. Thus, a freely diffusing fraction of DcuR-P is required for gene regulation.

Overall, the *in vitro* and *in vivo* data suggest a model where DcuS-P and DcuR, or DcuS and DcuR-P, respectively, form tight complexes at the membrane, where rapid adaptation of the DcuR phosphorylation state takes place. Upon phosphorylation, probably the affinity is decreased in the *in vivo* complex with full-length DcuS leading to DcuR-P release into the cytosol, where it can reach its target promoter by diffusion.

## MATERIALS AND METHODS

### Bacteria and molecular genetic methods.

All molecular genetic techniques were performed according to standard procedures (51). Genomic DNA was isolated and purified with the Nucleospin C+T Kit (Macherey & Nagel). Plasmids were isolated with the GenElute™ HP Plasmid Miniprep Kit (Sigma-Aldrich). E. coli strains were transformed with plasmids by electroporation (52). The purification and concentration of PCR products was performed with the GenElute™PCR Clean-Up Kit (Sigma-Alderich). DNA concentration was determined in UV microcuvettes with the BioPhotometer (Eppendorf). E. coli strains, plasmids, and primers utilized are listed in Table 2. All strains were grown aerobically in Luria Broth medium. Antibiotics were used at the following concentrations: 100 μg/mL ampicillin, 50 μg/mL kanamycin and 15 μg/mL tetracycline. The BACTH assay (28, 29) for *in vivo* DcuS/DcuR and PASC-Kin/DcuR interaction of T18 and T25 fusion proteins (Table 2) was performed as described in (17, 18).

**TABLE 2:** Strains of E. coli and plasmids used in this study

Strain of E. coli K12	Genotype or characteristics	Reference or source
BL21 (DE3)	fhuA2 [lon] ompT gal (λ DE3) [dcm] ΔhsdS with λ DE3 = λ sBamHIo ΔEcoRI-B int::(lacI::PlacUV5::T7 gene1) i21 Δnin5	(58)
C43 (DE3)	pLysS:F- ompT hsdSB(rB- mB-) gal dcm(DE3) pLysS (Cm^R^)	(53)
BTH101	F-, cya-99, araD139, galE15, galK16, rpsL1 (Strr), hsdR2, mcrA1, mcrB1	(28)
IMW691	MG1655, but *lacIZYA::frt, dcuR::kan^R^*	This work
Plasmids		
pUT18	Expression plasmid for C-terminal T18-fusions; derivative of pUC19 (Ap^R^)	(28)
pUT18C-zip	Expression plasmid for T18-Zip; derivative of pUT18C, (Ap^R^)	(28)
pKNT25	Expression plasmid for C-terminal T25-fusionens; derivative of pUC19 (Kan^R^)	(28)
pKT25-zip	Expression plasmid for T25-Zip; derivative of pKNT25, (Kan^R^)	(28)
pMW151	His6-DcuS expression plasmid, pET28a derivative (Kan^R^)	(21)
pMW266	His6-DcuR expression plasmid, pET28a derivative (Kan^R^)	(21)
pMW427	Expression plasmid for T25-DcuR; derivative of pKT25, (Kan^R^)	(27)
pMW428	Expression plasmid for DcuS-T18; pUT18 derivative, (Ap^R^)	(44)
pMW1076	Expression plasmid for PASc-Kin-T18 [DcuS(211-539)]; pUT18 derivative, (Ap^R^)	(17)
pMW1953	mYFP(A206K)-linker-DcuR expression plasmid (Apr, Tet^R^)	(26)
pMW2999	pMW428, but DcuS(H349A), (Ap^R^)	This work
pMW2902	pMW427, but DcuR(D56A), (Kan^R^	This work
pMW3020	pMW427, but DcuR(D56N), (Kan^R^)	This work
pMW3026	pMW764, but with *cfp*-*lacI* fusion and symmetric LacI binding site in front of *dcuB_P_*	This work
pMW3027	pMW1076, but DcuS(H349A), (Kan^R^)	This work
pMW2600	His6-PASc-Kin, (DcuS [AS 212-539]), pET28a derivative (Kan^R^)	This work

### Protein purification.

Proteins carrying N- or C-terminal His_6_ tag, His_6_-DcuR (pMW266), His_6_-eYfp-Linker-DcuR (pMW1953) and His_6_-PASc-Kinase (pMW2600) were produced in the BL21DE3 protein overproduction strain carrying the corresponding plasmids. Full-length His_6_-DcuS encoded by pMW151 was produced in strain C43 (DE3) (53). The strains were cultivated in LB medium at 30°C on a shaker (500 rpm) to OD_578nm_ of 0.5. Expression was induced with 1 mM isopropyl-β-d-thiogalactopyranoside (IPTG) for 4½ hours. The cells were harvested by centrifugation, washed and resuspended in buffer 1 (50 mM Tris/HCl, pH 7.7, and 10 mM MgCl_2_). The bacteria were broken by three passages through the French press (84 bar) in buffer 2 (50 mM Tris/HCl, pH 7.7, 10 mM MgCl_2_, 0.5 M NaCl and 10% glycerol). After removal of debris by centrifugation (8000 × *g*, 4°C) a cleared cell homogenate was obtained. The proteins His_6_-DcuR, eYfp-Linker-DcuR-His_6_ and PASc-Kin were purified from the cleared cell homogenate by Ni^2+^-nitrilotriacetic acid (NTA) agarose chromatography on a 3 mL column by gravity flow (elution buffer: 50 mM Tris/HCl, pH 7.7, 500 mM Imidazole, pH 7.7, 0.5 M NaCl, and 10% glycerol).

### Thermal denaturation.

The fluorescence-based thermal unfolding experiments were carried out with Prometheus NT.48 (NanoTemper Technologies). Samples of the proteins (freshly prepared, or after the SEC experiments, as indicated) were incubated at room temperature for 10 min at 1:1 molar ratio (45 or 55 μM) and loaded into 10 μL standard capillaries. The temperature was increased with a rate of 2°C/min from 20°C to 90°C and the intrinsic fluorescence emission of the tryptophans was measured at 330 nm and 350 nm. To correct for the intrinsic temperature dependence of fluorescence, the denaturation curves were constructed by plotting the ratio of the intensity measured at 350 and 330 nm, respectively.

### Size exclusion chromatography.

For the SEC experiments His_6_-PASc-Kinase (1 mL, 45 μM) and His_6_-DcuR (1 mL, 55 μM) were applied to HiLoad 16/600 Sephadex 200 (GE Healthcare) column employing a BioLogic DuoFlow F10 system (Bio-Rad) with an isocratic flow of 1 mL/min or 2 mL/min and pressure of 84 lb/in^2^ in buffer containing 50 mM Tris-HCl, 1 mM DTT, 50 mM KCl and 5 mM MgCl_2_ at pH 7.7 and 25°C. The elution profile was recorded at 280 nm. The eluate was collected in 1- or 2-mL samples. 20 μL of the collected samples were applied to SDS-PAGE and stained with Coomassie blue. Based on SEC experiments with protein standards (β-amylase MW 200 kDA, BSA MW 66 kDa, carbonic amylase MW 29 kDA and aprotinin MW 6.5 kDA) the relative molar mass (*M*_r_) of the samples was determined by linear regression. The contents of PAS_C_-Kin and DcuR in the peak fractions of the eluate fractions was determined by scanning and integration of the area and intensity of the stain in the SDS-PAGE by ImageJ. The relative molar contents of the proteins and their molar ratios were calculated from the protein staining intensity and the molar masses (18). Experiments were performed at least in triplicate (Fig. 2 and 4) or in duplicate (Fig. 5), and the molar contents and ratios are the means from three (or two) independent experiments (±SD).

### Microscale thermophoresis.

The concentration of the eYfp-Linker-DcuR construct was adjusted to 100 nM with MST buffer (Tris/HCl (pH 7.7), 10 mM MgCl_2_ and 2 mM DTT). The ligand PAS_C_-Kin was dissolved in MST buffer at a concentration of 200 μM and a series of 15 1:2-dilutions was prepared in the same buffer. For the measurement, each ligand dilution was mixed with one volume of labeled protein eYfp-Linker-DcuR. After 10 min incubation at 25°C followed by centrifugation at 10.000 × *g* for 3 min, the samples were loaded into standard Monolith NT.115 Capillaries (NanoTemper Technologies). The *K_d_* was determined after the distribution of labeled protein reached a steady state as indicated by a constant fluorescence signal. MST was measured in Monolith NT.115 instrument (NanoTemper Technologies) at 25°C. Instrument parameters were adjusted to 50% or 70% LED power and 20% MST power. 1:1 binding model was used for curve fitting in the steady state, with data normalization and baseline correction. Data of two independently pipetted measurements were analyzed, using the *MO Affinity Analysis* software version 2.2.3 (NanoTemper Technologies).

### Mass spectrometry and calculation of cellular contents of DcuR and DcuS.

Sixteen protein extracts (each four biological replicates of the following conditions: aerobic growth with or without fumarate, anaerobic growth with or without fumarate) from a previous study were used (47) to determine the protein content of DcuR in E. coli. Preparation of samples with 5 μg protein each was accomplished using an adapted SP3 bead-based protocol (54). In brief, proteins were reduced with 2.5 mM dithiothreitol for 30 min at 37°C and alkylated with 10 mM iodoacetamide for 15 min at 37°C in the dark, subsequently bound to the beads and washed with acetonitrile and ethanol prior to trypsin digestion in a protein to protease ratio of 25:1. Digestion was stopped with acetonitrile and beads were washed again. Finally, the peptides were released from the beads using 2% (vol/vol) DMSO in HPLC-grade water. After adding acetonitrile and acetic acid to a final concentration of 2% (vol/vol) and 0.1% (vol/vol), respectively, peptides were analyzed by nanoLC tandem mass spectrometry on an Orbitrap Exploris 480 mass spectrometer (Thermo Fisher Scientific) in data-independent mode. Data analysis was carried out in Spectronaut vs14.10.201222.47784 (Biognosys, Schlieren, Switzerland). Peptides were identified *via* the Direct-DIA algorithm (*sparse*) searching spectra against a protein database limited to E. coli K12 entries. Trypsin/P was set as enzyme, carbamidomethylation at cysteine as fixed and oxidation at methionine as variable modification. Quality control was applied on ion-level and a q-value <0.001 applied for further peptide identification and quantification. Normalization of intensities was carried out in Spectronaut and based on peptides identified in at least 50% of all samples. For calculation of the contents of DcuR (pmol × [mg cell protein]^−1^), the intensities of the precursor peptides that belong to the protein were summed together and divided by the number of theoretically observable peptides. This operation converts a measure that is expected to be proportional to mass (intensity) into one that is proportional to molar amount (iBAQ) (36, 55). Relative quantitation of DcuR between the four conditions investigated in this study was done on peptide level using R version 4.0.3. Methionine oxidized peptides were removed from the analysis before ratios between conditions were calculated using ROPECA statistics (56). Cellular amounts of DcuS were analyzed by SRM in a previous study in membrane fractions in three biological replicates (35). Here, the molar concentrations (μM*) of DcuR and DcuS per whole E. coli cell represents an estimate calculated from the contents (pmol × [mg cell protein]^−1^), assuming that the cell protein accounts to 5% of the cellular space (without taking into account uneven distribution or localization in the membrane), and a density of 1 g/mL for the cell and the proteins as a rough estimate. The concentration of the Dcus × DcuR complex (μM*) is calculated from law of mass action for complex formation from DcuS (μM*) and DcuR (μM*), and the *K_d_* values (1.38 μM in the absence of fumarate (PAS_C_-Kin × DcuR), and 0.028 μM in the presence of fumarate (PAS_C_-Kin × DcuR-P), compare Fig. 6). Experimental data and quantitative evaluation are presented in Table S1.

### Phosphorylation of proteins.

His_6_-DcuR or eYfp-Linker-DcuR (110 μM) were incubated for 1 h at 25°C with 50 mM carbamoyl-phosphate in phosphorylation buffer (50 mM Tris-HCl, pH 8. 5 mM MgCl_2_, 10 mM glycerol) (27). His_6_-PASc-Kinase (90 μM) was incubated for 10 min at 25°C with 1 mM ATP in phosphorylation buffer (50 mM Tris-HCl, pH 7.7, 1 mM DTT, 10 mM MgCl_2_).

### SMT.

SMT was done essentially as described in (57). In brief, DcuR-YFP molecules were tracked using 20 ms integration time, single molecule level was reached when single step bleaching events resulted in complete loss of fluorescence. A minimum of 5 steps was used for data analyses, which was performed using SMTracker version 2.0 (38). Displacements in x and y from three independent biological replicates were pooled and analyzed in a centered coordinate system. SD values are errors of derived from the fitting procedure, using “cross validation,” as described in (38). For a better comparison between cells grown with or without fumarate, Gaussian fitting was performed such that the best fitting was found for all data sets; thus, common diffusion constants were forced to present the best fit for both conditions.

### Data availability.

Data are presented in the published work and the Supplemental information.
