# Supplementary figures and images for: Tight Complex Formation of the Fumarate Sensing DcuS-DcuR Two-Component System at the Membrane and Target Promoter Search by Free DcuR Diffusion
Source: mSphere. 2022 Jul 7;7(4):e00235-22. doi: 10.1128/msphere.00235-22 (PMC9429925; doi:10.1128/msphere.00235-22)

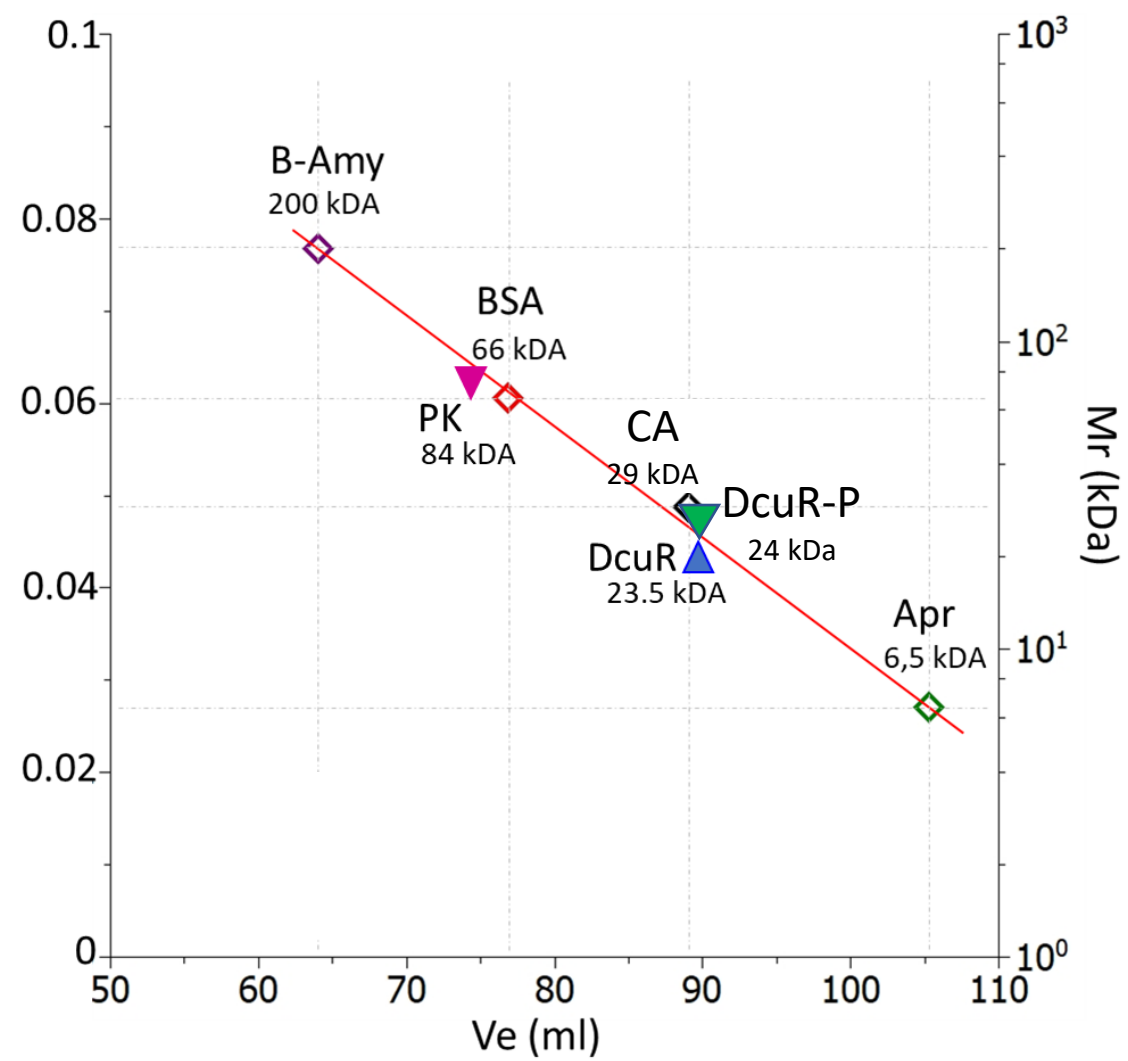

Supplement: FIG S3 [file msphere.00235-22-s0003.pdf]

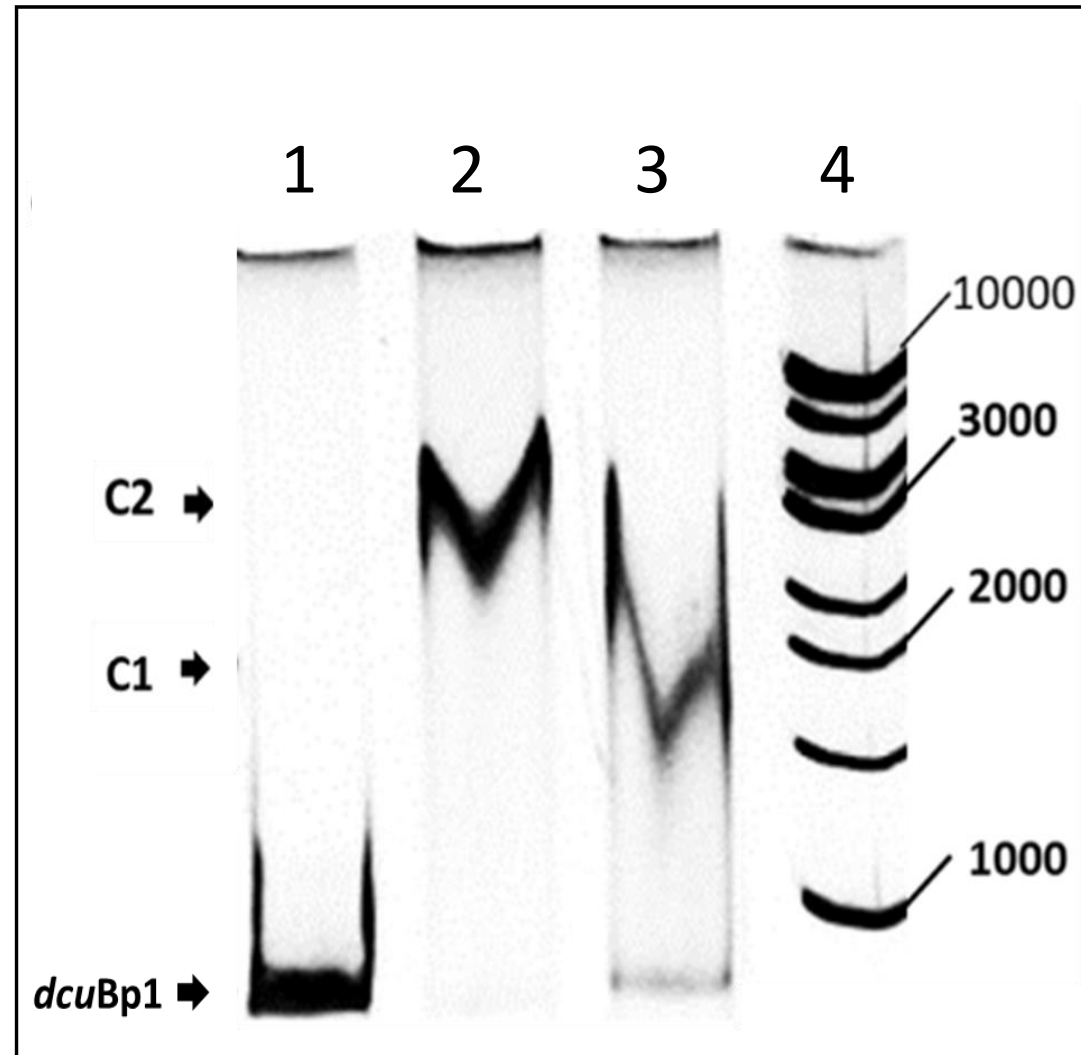

Supplement: FIG S2 [file msphere.00235-22-s0002.pdf]

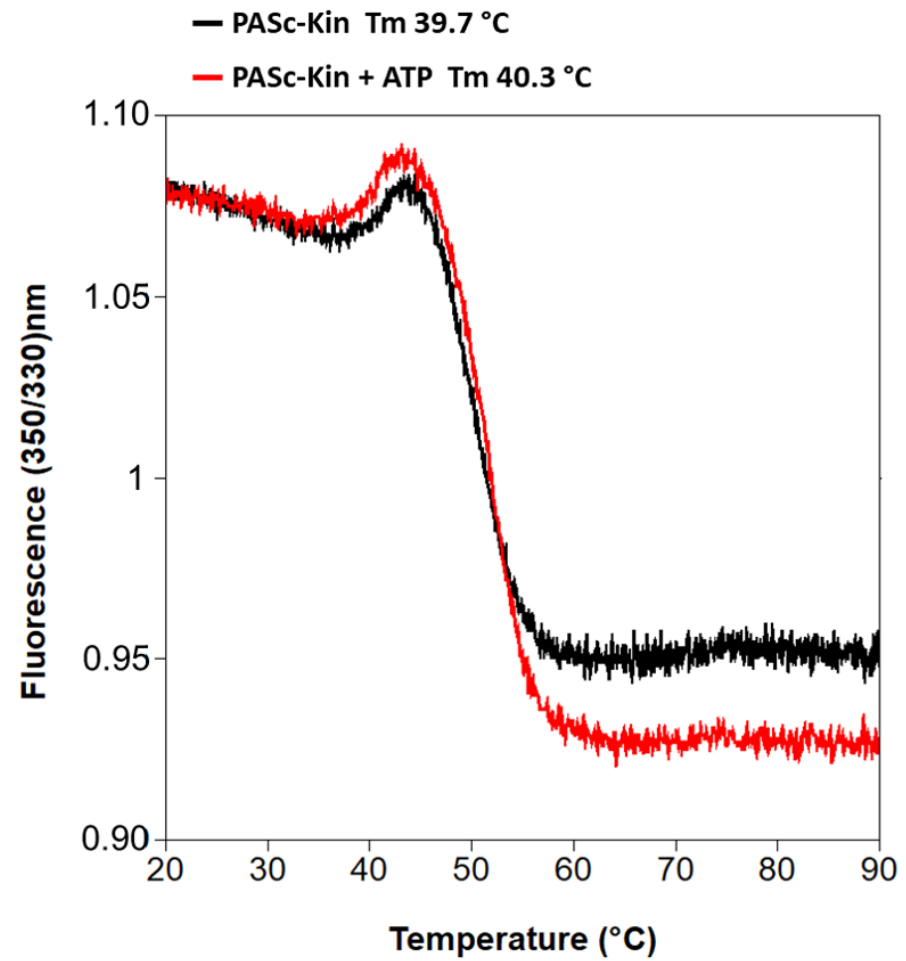

Supplement: FIG S6 [file msphere.00235-22-s0006.pdf]
