# Supplementary material for: Tight Complex Formation of the Fumarate Sensing DcuS-DcuR Two-Component System at the Membrane and Target Promoter Search by Free DcuR Diffusion
Source: mSphere. 2022 Jul 7;7(4):e00235-22. doi: 10.1128/msphere.00235-22 (PMC9429925; doi:10.1128/msphere.00235-22)
Supplement: FIG S1 [file msphere.00235-22-s0001.pdf]

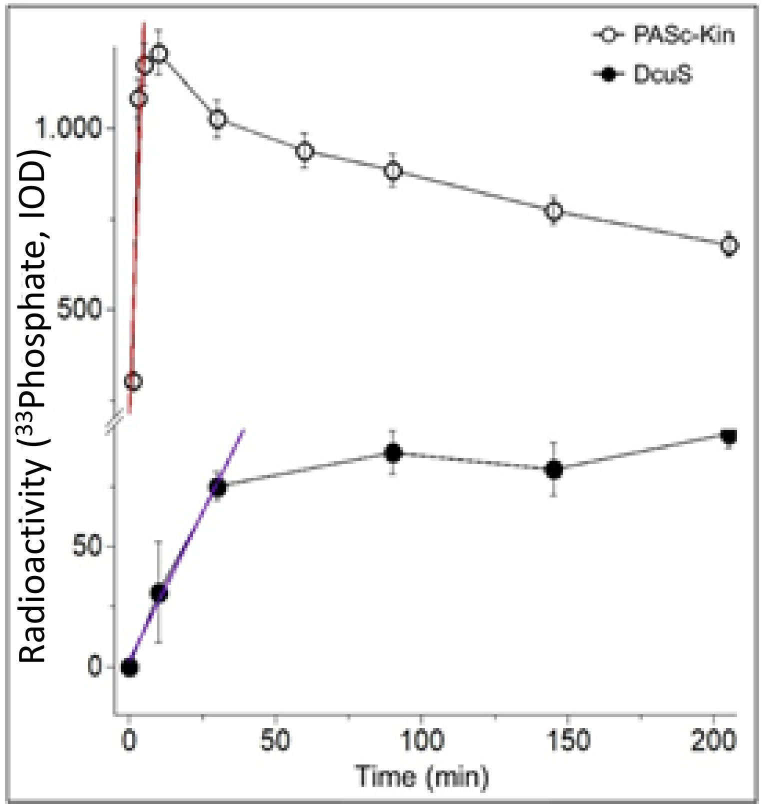

Autophosphorylation (data from figure)

	Reconstituted DcuS	PAS_C-Kin
**Protein concentration [µM]**	6.4	12
**Initial rate of phosphorylation (%/min)**	2.45	218
**t_{50} (min)**	45	1.5
