# Supplementary material for: Tight Complex Formation of the Fumarate Sensing DcuS-DcuR Two-Component System at the Membrane and Target Promoter Search by Free DcuR Diffusion
Source: mSphere. 2022 Jul 7;7(4):e00235-22. doi: 10.1128/msphere.00235-22 (PMC9429925; doi:10.1128/msphere.00235-22)
Supplement: FIG S5 [file msphere.00235-22-s0005.pdf]

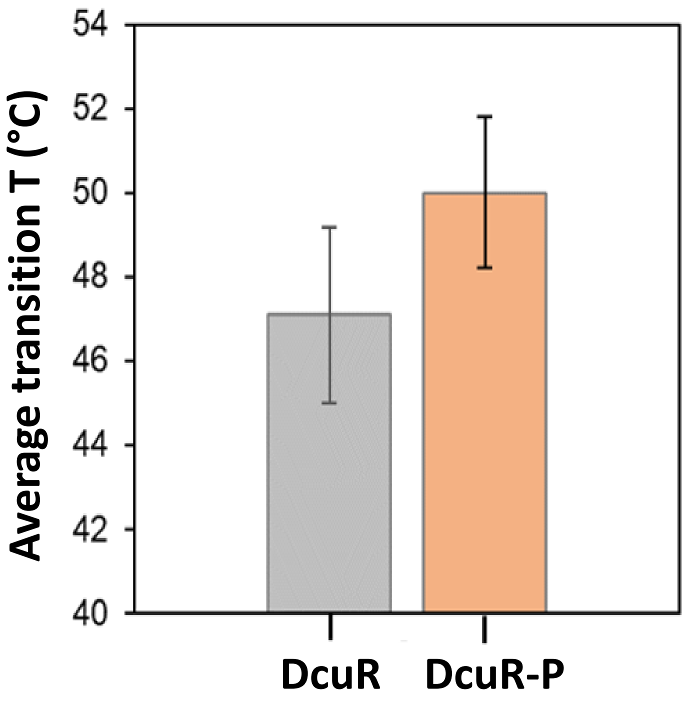

Tm value	DcuR: direct use after purification		DcuR: stored at -80°C before use		
Protein:	DcuR	DcuR + 50 mM CrbP	DcuR	DcuR + 50 mM CrbP	DcuR + 100 mM CrbP
Tm	48.4	52.5	44.9	48.1	48.9
Tm	49.3	52.2	45.8	49.1	49.3
mean	48.8	52.4	45.3	48.6	49.1
