# Supplementary material for: Tight Complex Formation of the Fumarate Sensing DcuS-DcuR Two-Component System at the Membrane and Target Promoter Search by Free DcuR Diffusion
Source: mSphere. 2022 Jul 7;7(4):e00235-22. doi: 10.1128/msphere.00235-22 (PMC9429925; doi:10.1128/msphere.00235-22)
Supplement: TABLE S1 [file msphere.00235-22-s0007.docx]

**Table S1: Estimation of DcuR levels from iBAQ values estimated in a global profiling approach (according to Krey et al., 2014).**

| **Sample** | **Replicate** | **Sum iBAQ (all proteins)** | **iBAQ DcuR** | **ng DcuR in injected sample** | **M DcuR ng/mol** | **nmol/g** | **pmol/mg** | **pmol/mg (Mean)** | **g protein/**  **cell** | **g protein/**  **cell** | **g/cell (Mean)** | **molecules/**  **cell** | **molecules/ cell (Mean)** |
| --- | --- | --- | --- | --- | --- | --- | --- | --- | --- | --- | --- | --- | --- |
| Aerobic | 1 | 1.1E+09 | 5.1E+03 | 0.007 | 2.75E+13 | 0.169 | 169 |  | 4.5E-13 | 1.3E-13 |  | 14 |  |
| Aerobic | 2 | 1.1E+09 | 3.6E+03 | 0.005 | 2.75E+13 | 0.123 | 123 |  | 4.5E-13 | 1.3E-13 |  | 10 |  |
| Aerobic | 3 | 1.0E+09 | 9.5E+03 | 0.014 | 2.75E+13 | 0.341 | 341 | **197** | 4.8E-13 | 1.5E-13 | **1.4E-13** | 30 | **17** |
| Aerobic | 4 | 1.0E+09 | 4.4E+03 | 0.006 | 2.75E+13 | 0.154 | 154 |  | 4.8E-13 | 1.4E-13 |  | 13 |  |
| plus fumarate | 1 | 1.1E+09 | 2.2E+04 | 0.031 | 2.75E+13 | 0.742 | 742 |  | 3.3E-13 | 1.0E-13 |  | 44 |  |
| plus fumarate | 2 | 1.1E+09 | 2.8E+04 | 0.039 | 2.75E+13 | 0.941 | 941 |  | 3.2E-13 | 9.7E-14 |  | 55 |  |
| plus fumarate | 3 | 1.1E+09 | 2.6E+04 | 0.036 | 2.75E+13 | 0.885 | 885 | **830** | 3.7E-13 | 1.1E-13 | **1.0E-13** | 60 | **50** |
| plus fumarate | 4 | 1.1E+09 | 2.4E+04 | 0.031 | 2.75E+13 | 0.754 | 754 |  | 3.2E-13 | 9.5E-14 |  | 43 |  |
| Anaerobic | 1 | 9.9E+08 | 1.8E+04 | 0.027 | 2.75E+13 | 0.652 | 652 |  | 5.4E-13 | 1.6E-13 |  | 63 |  |
| Anaerobic | 2 | 1.1E+09 | 2.1E+04 | 0.028 | 2.75E+13 | 0.673 | 673 |  | 5.2E-13 | 1.6E-13 |  | 63 |  |
| Anaerobic | 3 | 1.0E+09 | 1.5E+04 | 0.021 | 2.75E+13 | 0.519 | 519 | **629** | 5.0E-13 | 1.5E-13 | **1.6E-13** | 47 | **59** |
| Anaerobic | 4 | 1.0E+09 | 1.9E+04 | 0.028 | 2.75E+13 | 0.672 | 672 |  | 5.2E-13 | 1.6E-13 |  | 63 |  |
| plus fumarate | 1 | 1.1E+09 | 2.8E+04 | 0.038 | 2.75E+13 | 0.933 | 933 |  | 2.9E-13 | 8.7E-14 |  | 49 |  |
| plus fumarate | 2 | 1.2E+09 | 2.8E+04 | 0.035 | 2.75E+13 | 0.848 | 848 |  | 3.6E-13 | 1.1E-13 |  | 55 |  |
| plus fumarate | 3 | 1.1E+09 | 3.3E+04 | 0.045 | 2.75E+13 | 1.088 | 1088 | **979** | 2.9E-13 | 8.8E-14 | **9.5E-14** | 58 | **56** |
| plus fumarate | 4 | 1.1E+09 | 3.1E+04 | 0.043 | 2.75E+13 | 1.048 | 1048 |  | 3.2E-13 | 9.7E-14 |  | 61 |  |
