# Supplementary material for: Tight Complex Formation of the Fumarate Sensing DcuS-DcuR Two-Component System at the Membrane and Target Promoter Search by Free DcuR Diffusion
Source: mSphere. 2022 Jul 7;7(4):e00235-22. doi: 10.1128/msphere.00235-22 (PMC9429925; doi:10.1128/msphere.00235-22)
Supplement: FIG S4 [file msphere.00235-22-s0004.pdf]

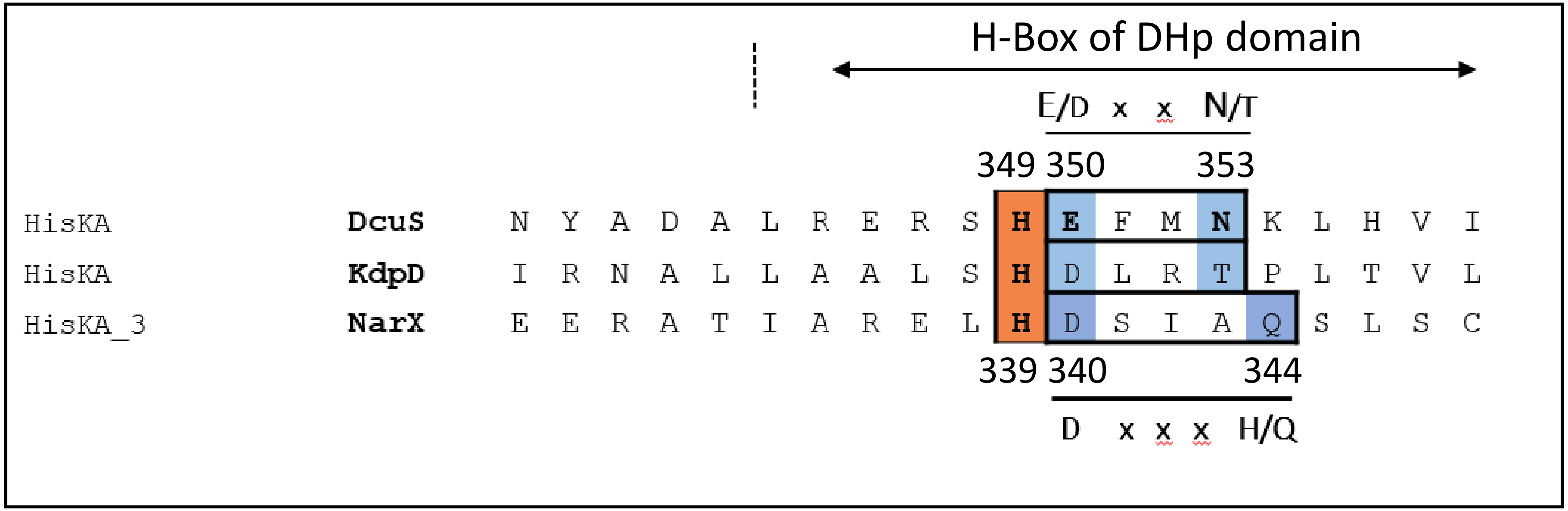
H-Box of DHp domain
E/D x x N/T
349 350 353
HisKA DcuS N Y A D A L R E R S H E F M N K L H V I
HisKA KdpD I R N A L L A A L S H D L R T P L T V L
HisKA_3 NarX E E R A T I A R E L H D S I A Q S L S C
339 340 344
D x x x H/Q
